# Metal oxide nanoparticles for acne vulgaris: mechanisms, synthesis, formulation, and translational prospects

**DOI:** 10.1039/d6ra04926e

**Published:** 2026-09-01

**Authors:** Akhil Nair, Sindhoor S. M., Shravya D. Rai, Gladyston Netto, Srinivas Mutalik, Pavithra Pradeep Prabhu

**Affiliations:** a Nitte (Deemed to be University), NGSM Institute of Pharmaceutical Sciences (NGSMIPS), Department of Pharmaceutics Mangalore India; b Dnyaan Prasad Global University School of Pharmacy and Research Pimpri Pune India; c Dr D. Y. Patil Unitech Society's, Dr D. Y. Patil Institute of Pharmaceutical Sciences and Research Pimpri Pune India; d Department of Pharmaceutics, Manipal College of Pharmaceutical Sciences, Manipal Academy of Higher Education Manipal Karnataka 576104 India; e Department of Pharmacognosy, Manipal College of Pharmaceutical Sciences, Manipal Academy of Higher Education Manipal Karnataka 576104 India

## Abstract

Acne vulgaris is a chronic inflammatory condition of the pilosebaceous unit resulting from follicular hyperkeratinization, increased sebum production, overgrowth of *Cutibacterium acnes*, and immune activation, with subsequent activation of Toll-like receptor signalling and increased expression of IL-1β, IL-8, and matrix metalloproteinases, leading to scarring and hyperpigmentation. Conventional treatments such as topical retinoids, benzoyl peroxide, antibiotics, oral isotretinoin, and hormonal therapies are often associated with limited efficacy, slow response, irritation, photosensitivity, and poor tolerability, prompting the search for non-antibiotic strategies that preserve the microbiome. This review evaluates metal oxide nanoparticles as multifunctional agents with acne-targeted, anti-inflammatory, antioxidant, and photocatalytic activities suitable for the follicular microenvironment. The emphasis is placed on zinc oxide, titanium dioxide, copper oxide, iron oxide, magnesium oxide, cerium oxide, aluminium oxide and manganese oxide nanoparticles. The review further examines synthesis methods (physical, wet-chemical, and green plant/microbial synthesis), formulation platforms (hydrogels/nanogels, electrospun fibers, lipid carriers, microneedles, and near-infrared (NIR)-responsive nanomotors), as well as safety and regulatory aspects. Available evidence suggests that primary particle size (typically 20–70 nm), hydrodynamic aggregation (200–700 nm for follicular depots), zeta potential (−10 to −30 mV), and surface coatings (*e.g.*, hyaluronic acid, polysaccharides, and polyethylene glycol (PEG)) significantly influence dermal fate. Metal oxide nanoparticles represent promising adjuvants and potential alternatives for the treatment of mild-to-moderate acne vulgaris and maintenance therapy, provided they are synthesized using standardized protocols, formulated through microbiome-friendly approaches, toxicologically evaluated using harmonized assessment frameworks, and validated through rigorous, adequately powered clinical trials.

## Abstract 2

This review evaluates metal oxide nanoparticles as multifunctional agents for acne, highlighting their antimicrobial, anti-inflammatory, antioxidant and photocatalytic activities, synthesis methods, formulation platforms, safety and regulatory aspects.

## Graphical abstract

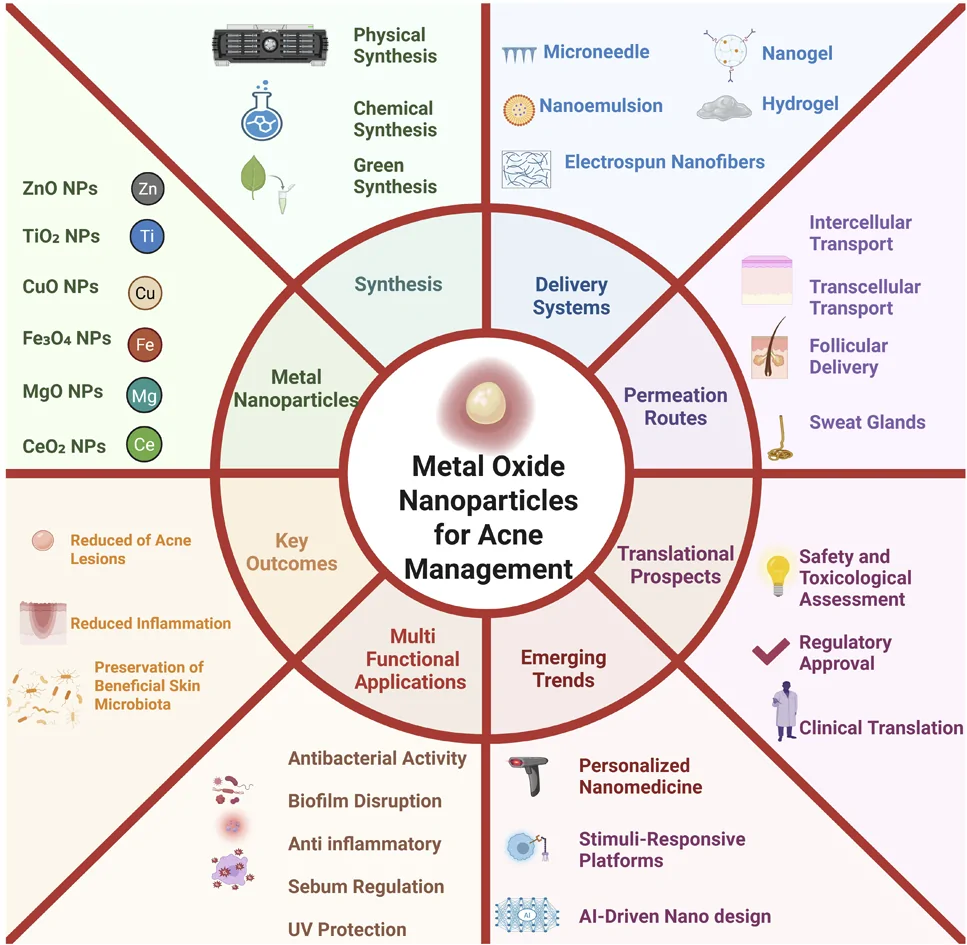

## 1. Introduction

Acne vulgaris is a chronic inflammatory disease of the pilosebaceous unit, characterized by four key pathogenic factors: hyperkeratinization of pilosebaceous follicles, increased sebum production, microbial dysbiosis, and immune activation. Acne is not life-threatening, but it can last for years and may lead to sequelae such as scarring and post-inflammatory hyperpigmentation, causing significant psychosocial consequences for patients.^1^ The key role of *Cutibacterium acnes* in disease pathogenesis is that it proliferates in the anaerobic follicular microenvironment and activates pro-inflammatory signalling by binding and stimulating Toll-like receptors, which results in the upregulation of interleukin (IL)-1β, IL-8, and matrix metalloproteinases. These mediators attract neutrophils and macrophages to the areas of follicular rupture.^2^ In addition to microbial factors, genetic predisposition, androgen-induced overactivity of the sebaceous glands, environmental exposures, and lifestyle factors are modulators of acne severity and influence the disease phenotype, chronicity, and clinical outcomes.^3^

Current acne treatments include topical retinoids, benzoyl peroxide (BPO), azelaic acid, salicylic acid, antibiotics, oral isotretinoin, and certain hormonal therapies.^4^ Although many interventions are effective, their use is frequently limited by adverse effects such as irritation, burning, itching, stinging, erythema and photosensitivity.^5^ Furthermore, the use of antibiotic-based regimens presents an additional challenge, as prolonged or repeated use of antibiotics on the skin, topically or systemically, can result in resistance of *Cutibacterium acnes* and other microorganisms of the skin, reducing the efficacy of the treatment and raising a significant public health concern. As a result, alternative non-antibiotic strategies to inhibit bacterial overgrowth while preserving the commensal skin microbiome are gaining great interest.^6^

To address these unmet clinical needs, nanotechnology has emerged as a promising strategy for improving dermal delivery, increasing the local concentration of drugs, and enabling controlled and stimulus-responsive release at the site of disease.^7^ Metal oxide nanoparticles (MONPs) are of special interest in this context because of their small size (usually 1–100 nm), high surface area-to-volume ratio, redox activity, and surface chemistry that can be tailored to the application.^8^ These properties have the potential to either adsorb molecules onto their surface or encapsulate them within their core, which makes it easier to precisely transport and distribute active ingredients to biological sites.^9^ Their anti-inflammatory, antioxidant, and photocatalytic properties are all multifaceted, which is beneficial in acne, where the pathogenic process is mostly confined to the follicular and perifollicular microenvironment of the pilosebaceous follicles.^10^

The metal oxide systems studied for acne-related applications are mainly zinc oxide (ZnO), titanium dioxide (TiO_2_), copper oxide (CuO), magnetite (Fe_3_O_4_), maghemite (γ-Fe_2_O_3_), magnesium oxide (MgO), cerium oxide (CeO_2_), manganese oxide (MnO_2_), and aluminium oxide (Al_2_O_3_).

**Fig. 1:**
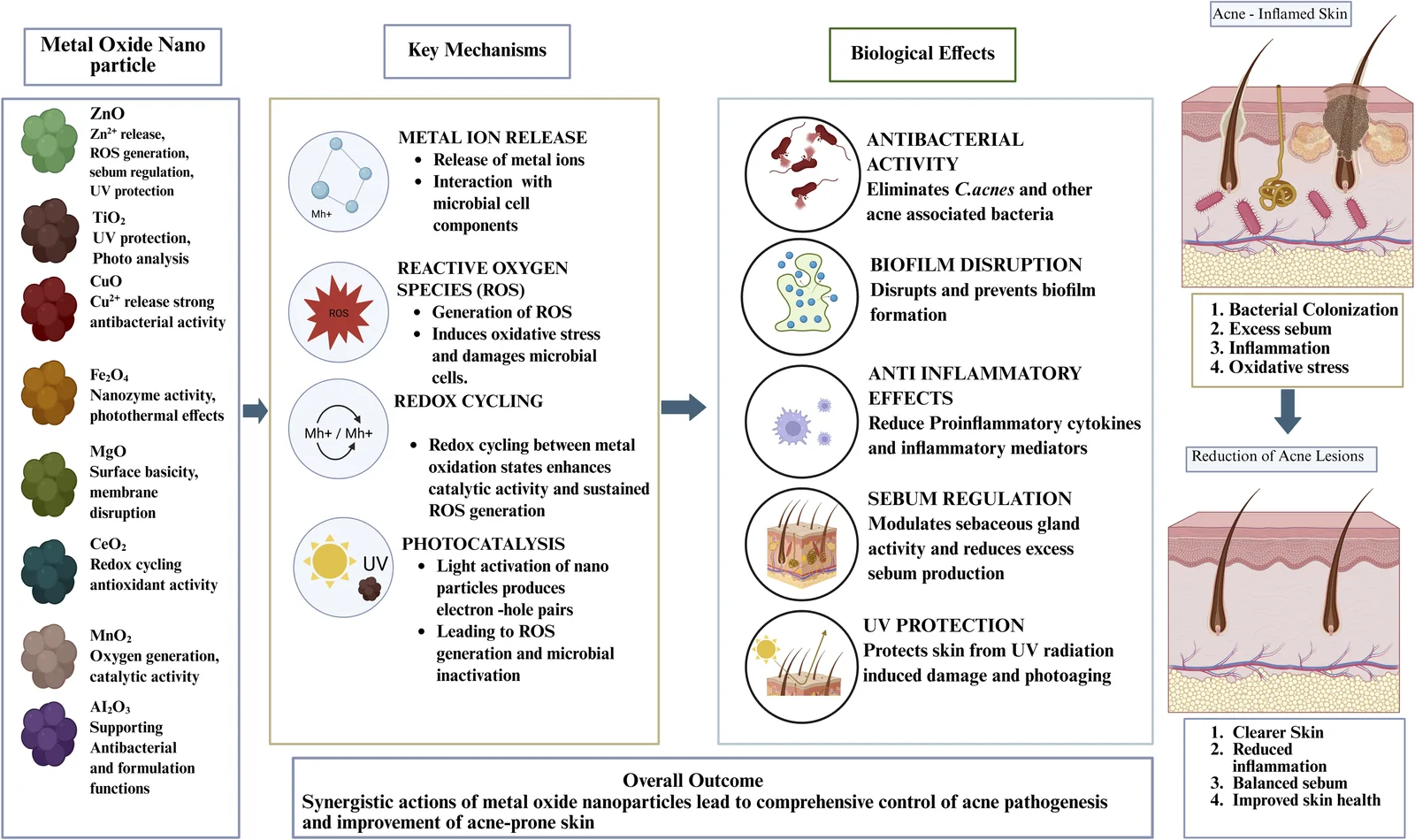
Biological mechanisms of metal oxide nanoparticles in acne therapy and their therapeutic effects. This image was created using BioRender software (https://www.biorender.com/).

ZnO has been widely studied because of its wurtzite crystal structure, broad UV-blocking properties, and sustained release of Zn^2+^ ions, which disrupt bacterial membranes and inhibit enzyme activity.^11^ TiO_2_ is valued for its photoprotective properties and, particularly in its anatase polymorph, its ability to generate reactive oxygen species (ROS) upon UV irradiation, thereby inactivating bacteria.^12^ CuO and Cu_2_O nanoparticles (NPs) exhibit p-type semiconducting behavior and copper redox cycling, which underlie their strong contact-killing activity,^13,14^ while Fe_3_O_4_ NPs display nanozyme-like peroxidase and catalase activity alongside magnetic responsiveness.^15^ The bactericidal mechanism of MgO operates through surface basicity and mechanical disruption of the bacterial membrane,^16^ whereas that of CeO_2_ operates through reversible Ce^3+^/Ce^4+^ redox chemistry, scavenging ROS and reducing oxidative-stress-mediated inflammation.^17^ The crystal structure, defect density, and ion-release kinetics of each oxide critically influence the particle morphology, ROS generation, and overall dermatological performance.^18^ The major mechanisms involved in MONP-mediated acne therapy are shown in Fig. 1.

One of the key advantages of MONPs over conventional antibiotics is their ability to hinder bacterial growth through multiple concurrent mechanisms, including disruption of the cell membrane, induction of intracellular oxidative stress, destabilization of biofilms, and metal ion-mediated toxicity.^19^ This multi-target mode of activity substantially reduces the possibility of stable resistance development compared to single-target antibiotics and supports the use of MONPs as non-antibiotic or antibiotic-sparing therapeutic agents.^20^

Importantly, the selective suppression of pathogenic *Cutibacterium acnes* while preserving the commensal skin microbiome remains difficult to achieve with broad-spectrum topical antibiotics, making rational particle engineering an attractive option.^21^

However, the stratum corneum (SC) is a formidable barrier to NP transport, and the extent of skin penetration is strongly influenced by the physicochemical properties of NPs.^22^ Following topical application, MONPs have been reported to access the skin through three principal pathways: (i) intercellular diffusion through the lipid lamellae of the SC, (ii) transcellular diffusion across the corneocytes, and (iii) the follicular pathway (transappendageal) through the openings of hair follicles.^23^ The follicular route is of particular interest for acne therapy because hair follicles are natural reservoirs for *Cutibacterium acnes* colonization and can retain particles up to approximately 700 nm in diameter.^24^ Thus, particle size, shape, surface charge, hydrophobicity, and coating chemistry are important factors that dictate the path and depth of penetration into the skin.^24^

The synthesis route of MONPs significantly affects their crystallinity, defect density, morphology, and surface functionality, all of which control the therapeutic performance of the nanoparticles. Laser ablation and vapor-phase deposition are physical techniques that result in very pure nanoparticles, but are associated with challenges in controlling the size and shape of the particles in large-scale production.^25^ In contrast, wet chemical methods such as hydrothermal synthesis, co-precipitation, sol–gel processing, and combustion synthesis are widely employed because they provide greater control over particle size, morphology, crystallinity, and surface properties.^26,27^ Among the green synthesis techniques, those that use plant or microbe-based capping agents are more interesting for dermatological applications because they provide intrinsic bioactivity, enhance aqueous colloidal stability, and have a low environmental footprint.^28^ Biogenic coronas can also enhance the tolerability and support the long-term performance of topical acne formulations.^29,30^

Recent developments have transformed MONPs into multifunctional therapeutic platforms.^31^ Notable examples include the use of ZnO-containing hydrogels and electrospun nanofibers,^32^ CuO-loaded composite films,^33^ Fe_3_O_4_-based nanocomposites for photothermal or magnetically guided treatment,^34^ and MnO_2_-based microneedle systems designed to eradicate biofilms within the follicular canals.^10^ TiO_2_ continues to be significant not only as a UV filter in sun creams designed for acne-prone skin but also as a light-activated antibacterial agent.^12,35^ In addition, the superoxide dismutase and catalase-mimetic activities of CeO_2_, together with its antioxidant and anti-inflammatory effects in models of inflamed skin, may provide a means of inhibiting the oxidative cascade that perpetuates follicular inflammation.^36^ Collectively, these developments suggest that future acne pharmacotherapy may evolve towards multifunctional, locally acting oxide nanoplatforms capable of targeting multiple pathogenic pathways simultaneously.^37^

Several recent reviews have examined nanotechnology for acne; however, the scope does not cover material chemistry of metal oxides. Mustaffa *et al.* surveyed nanoformulation strategies and the delivery barriers imposed by the pilosebaceous unit. Their analysis focused on organic carriers such as lipid nanoparticles, vesicular systems, and polymeric platforms rather than inorganic oxide cores.^21^ Kelidari *et al.* conducted a PRISMA systematic review of clinical evidence for topical nano-based delivery in acne. Although that work is valuable for evaluating clinical outcomes, the included products are carrier systems loaded with conventional actives; therefore, the redox and ion-release chemistry that distinguishes one oxide from another falls outside its scope.^38^ Crainic *et al.* provided a focused systematic review of ZnO NPs, which is complementary to the present work but is restricted to a single oxide.^11^ A broader dermatological overview by Chen *et al.* discussed nanomedicine in acne at a general level.^37^ By linking the crystal structure, defect density, and ion release kinetics to anti *Cutibacterium acnes* activity, anti-inflammatory effects, biofilm disruption, and skin penetration, this work aims to fill the gap between broad nanocarrier-centric surveys and mechanistically driven, oxide-specific analysis.^11^

This review addresses this gap by comparing eight oxide systems (ZnO, TiO_2_, CuO, Fe_3_O_4_/γ-Fe_2_O_3_, MgO, CeO_2_, MnO_2_ and Al_2_O_3_) on a common mechanistic and translational basis and distinguishes oxides with genuine acne-specific evidence from those supported only by general antibacterial data. We further emphasize acne-relevant formulation platforms, including nanogels, hydrogels, nanofibers, microneedles, and nanomotors, together with the clinical translational aspects of these oxide systems, thereby providing a focused framework for the rational design of MONP-based therapies for acne vulgaris. Table 1 summarizes the differences between the scope of the present review and those of these recent articles. This review aims to provide a framework for the rational design of effective, safe, and microbiome-conscious MONP-based therapies for acne management.^21^

**Table 1:** Scope of present review compared with previous review

Scope	Nanomaterials covered and distinctive focus of previous reviews	References
Nanoformulation strategies and follicular delivery barriers in acne	Lipid, vesicular and polymeric carriers. Organic carrier centred	21
Review of topical nano-based delivery in acne	Carrier systems loaded with conventional actives. Emphasizes clinical outcomes of carrier systems	38
Review of ZnO nanoparticles for acne	Zinc oxide only. Focused on ZnO	11
This study systematically summarizes the research landscape and developmental trends of nanomaterials in acne treatment	Solid lipid nanoparticles, nanostructured lipid carriers, nanoemulsions, liposomes, niosomes, ethosomes, transfersomes, polymeric nanoparticles, microsponges	37
Structure–activity, follicular delivery, oxide-specific safety and translation for acne	Eight oxides: ZnO, TiO_2_, CuO, Fe_3_O_4_/γ-Fe_2_O_3_, MgO, CeO_2_, MnO_2_, Al_2_O_3_. Integrates crystal structure and ion-release chemistry with *Cutibacterium acnes* activity, follicular delivery and oxide-specific safety in a comparative framework	Present review

## 2. Mechanisms of skin permeation of MONPs

**Fig. 2:**
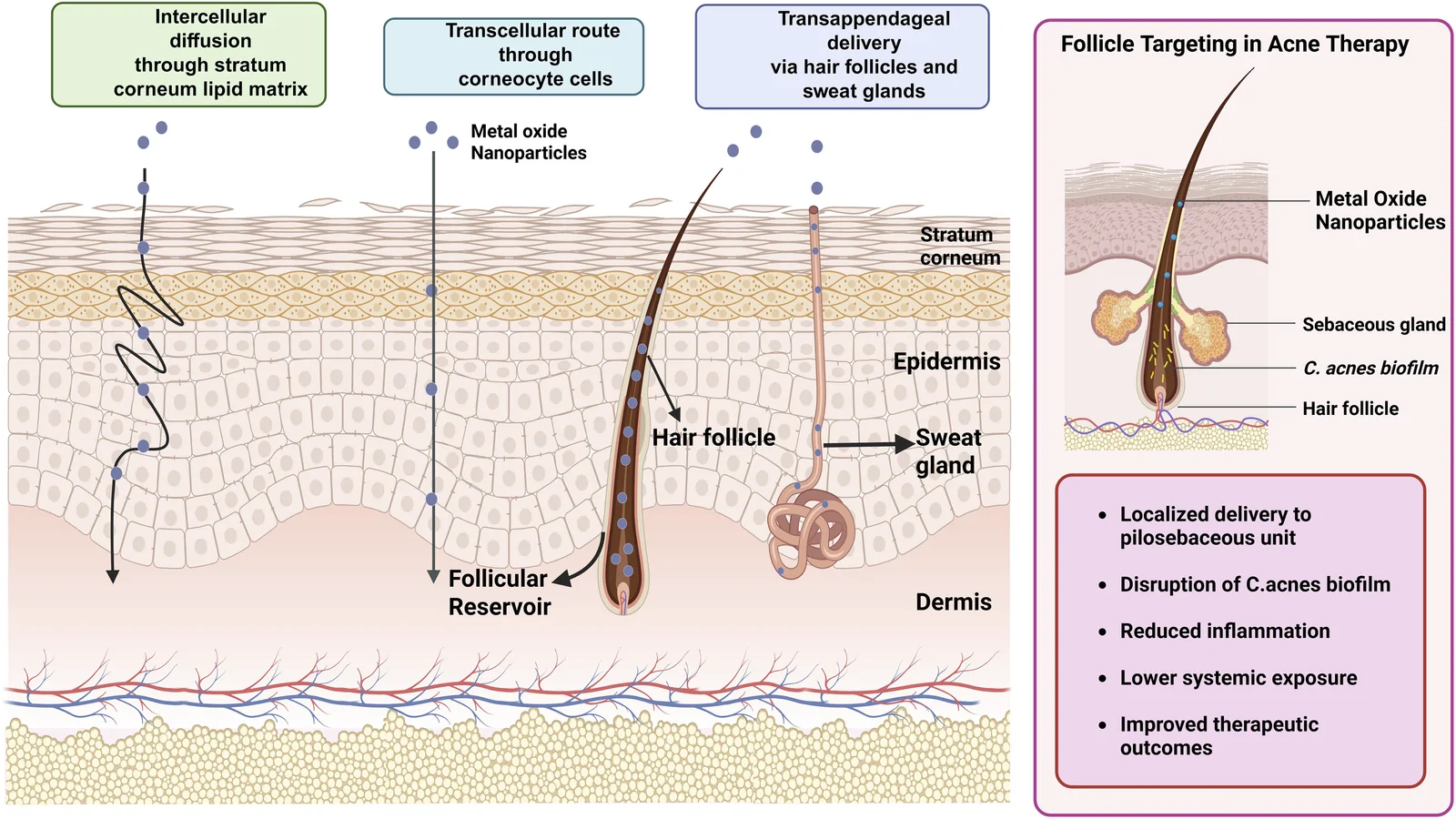
Schematic representation of principal skin permeation pathways for metal oxide nanoparticles. This image was created using BioRender software (https://www.biorender.com/).

MONPs interact with the skin barrier through multiple parallel pathways, and their transport is governed by a combination of particle size, surface chemistry, and microanatomy of the stratum corneum and skin appendages.^24^ As illustrated in Fig. 2, MONPs can permeate the skin through three principal pathways: intercellular diffusion through the lipid matrix of the stratum corneum, transcellular transport across corneocytes, and transappendageal delivery *via* hair follicles and sweat glands.^23^ Efficient access to the follicular infundibulum without systemic exposure is especially beneficial for acne therapy, as *Cutibacterium acnes* colonization and the associated inflammatory microenvironment are concentrated within pilosebaceous units.^21^

Passive diffusion across the stratum corneum occurs mainly through the intercellular lipid matrix, which is a tortuous route composed of ceramides, cholesterol, and free fatty acids.^39^ Molecules that are very small (<500 Da) and have moderate lipophilicity can traverse this route efficiently,^40^ whereas MONPs are largely confined to the outer layers and openings of hair follicles.^9^ Both zinc oxide (ZnO) and titanium dioxide (TiO_2_) NPs in the 20–100 nm size range have been shown to consistently localize in the superficial portions of the stratum corneum, skin folds, and follicular openings in *ex vivo* and *in vivo* studies of human skin, with little or no evidence of intact particle penetration into the viable epidermis or dermis under normal conditions.^41^ Confocal and multiphoton microscopy, together with electron microscopy, showed that ZnO NPs are deposited as a quasi-continuous mineral layer at the surface of the stratum corneum, whereas ionic zinc species generated by slow dissolution can permeate into the viable epidermis.^42^

Although the intercellular and transcellular pathways mainly contribute to surface deposition in the stratum corneum, the follicular (transappendageal) route is particularly important for acne-targeted MONPs because hair follicles represent a structural discontinuity in the skin barrier and act as reservoirs that can be repeatedly accessed through the movement of hair shafts.^23,43^ Quantitative tape-stripping and cyanoacrylate biopsy studies have demonstrated that nanoparticulate formulations accumulate in follicular casts to depths ranging from several hundred micrometres to approximately 1.5 mm, depending on the particle size and vehicle composition.^44^ Hair follicle targeting studies indicate that particles in the 400–700 nm range tend to be retained within the follicular duct,^45^ whereas smaller particles (20–40 nm) progressively migrate from follicles into the surrounding viable tissue over time,^45^ highlighting the possibility of tuning iron oxide nanoparticles to either favor depot formation or deeper perifollicular penetration.^46^

In addition to passive diffusion, some active and quasi-active processes are involved in MONP-skin interactions at the cellular level.^22^ Once the MONPs reach the viable layers, they may be internalized by keratinocytes, Langerhans cells, and dermal immune cells through clathrin- or caveolae-mediated endocytosis, driven by electrostatic interactions and receptor recognition of adsorbed protein coronas.^47^*In vitro* studies with model metallic NPs have shown that smaller (<50 nm), positively charged particles exhibit greater uptake by immune cells and induce stronger cytokine responses than their larger or neutrally charged counterparts.^48^ This observation supports the use of surface coatings in acne formulations that are either mildly anionic or neutral (such as hyaluronic acid (HA) and polysaccharides) to reduce off-target immunostimulation while maintaining antibacterial activity.^49^

These permeation pathways are greatly affected by physicochemical parameters.^50^ The 10–100 nm size range is deemed ideal for achieving a high surface area and strong antibacterial activity with minimal systemic penetration.^51^ Many ZnO and iron oxide NPs in topical products fall within the 20–70 nm range.^52^ The zeta potential strongly influences the colloidal stability of nanoparticles and their interactions with negatively charged skin surfaces.^11^ Mildly anionic systems, such as many formulations of ZnO and cerium oxide (CeO_2_), are generally associated with reduced nonspecific protein adsorption and lower keratinocyte toxicity,^53,54^ whereas highly cationic systems, such as MgO and Al_2_O_3_ may exhibit greater affinity for the skin surface in addition to being associated with a higher risk of irritation at comparable doses.^55,56^

Hydrophobicity and surface functionalization (*e.g.*, PEGylation, lipid, or polysaccharide coatings) also influence whether particles are retained within the stratum corneum, partition into the follicular sebum, or penetrate the viable layers of the skin. For example, PEGylated silica NPs have been shown to penetrate more deeply into hair follicles than thiolated silica NPs, illustrating how surface chemistry can be exploited to enhance follicular targeting.^57^

Quantitative information on the interaction of MONP with the skin can be obtained using Franz diffusion cells, tape stripping, and confocal Raman or multiphoton microscopy.^22^ Franz diffusion studies with ZnO NPs have shown that, although intact particles remain largely in the donor compartment and superficial stratum corneum, they can enhance the permeation of co-formulated solvents and active compounds into the skin. An increase of 20–30% in cumulative permeation has been reported for several hydrophilic and lipophilic probes, likely due to subtle perturbation of stratum corneum lipid packing.^58^ Confocal imaging of fluorescent NPs applied to human or porcine skin indicates that intact particles typically remain within the outer 10–20 µm of the stratum corneum, whereas hair-follicle targeting strategies and mechanical stimulation (*e.g.*, massage, hair movement) promote deeper transappendageal delivery.^24^ Raman depth profiling and elemental mapping further confirmed that, in ZnO sunscreen formulations, zinc detected in the viable epidermis is predominantly present as dissolved ions rather than as particulate ZnO, even under barrier-impaired or occluded conditions.^42^

Active delivery strategies can be integrated with conventional permeation pathways through follicle-targeted systems, such as microneedles, nanofibers, and Janus nanomotors.^59,60^ Zn-peroxide/MnO_2_ Janus nanomotors encapsulated within HA microneedles can mechanically penetrate the skin and, upon irradiation with 808 nm near-infrared (NIR) light, self-propel through dense *Cutibacterium acnes* biofilms while generating ROS and oxygen locally. These systems are a step towards actively navigated MONP delivery, where particle propulsion, chemotaxis, and biofilm penetration are important design parameters in addition to particle size and surface charge.^10^

The dermal fate of MONPs depends on their primary particle size, hydrodynamic diameter, and zeta potential, which are largely affected by the functional route and surface functionalization.^61^ Engineering NPs with size distributions in the range of 20–700 nm, surface coatings, and morphologies are required for follicular targeting, along with precise control over nucleation and growth conditions.^62^

### 2.1 Delivery across the sebaceous follicular plug

One potential limitation of topical oxide formulations is that the acne follicle is occluded by a lipid-rich sebum and corneocyte plug.^63^ In contrast, most metal oxide nanoparticles possess polar, high-surface-energy surfaces that are poorly wetted by the lipid-rich sebum.^64,65^ Consequently, they tend to stall at the follicular orifice or partition into the aqueous vehicle rather than into the plug. Therefore, effective follicular delivery depends as much on formulation design as on nanoparticle properties and several complementary strategies have been developed to address this challenge.^66^

Coating oxides with lipids, phospholipids, or amphiphilic polymers increases sebum compatibility. The same effect follows from embedding oxides in solid lipid or nanostructured lipid carriers, which favors partitioning into the follicular lipid.^67^ Lipid particles in the 200–400 nm range accumulate efficiently in follicles, and their charge and hydrophobicity can be tuned to localize in the sebaceous region.^40^ PEGylated silica nanoparticles penetrated deeper into the hair follicles than the thiolated silica. These findings demonstrate that surface chemistry, in addition to particle size, plays a critical role in determining the depth of follicular penetration.^57^

A complementary strategy involves chemical conditioning of the follicular plug itself. Salicylic acid is a lipophilic beta-hydroxy acid that is used as a standard acne treatment. It is comedolytic and loosens the corneocyte lipid plug.^68,69^ Low levels of surfactants also reduce the interfacial tension between an aqueous particle dispersion and sebum.^70^ Co-formulating oxides with these agents improves access to the follicular canal while maintaining each agent at sub-irritant concentrations.^21^

Particles engineered to the 400–700 nm follicular-retention window form depots in the duct, and massage or hair movement drives them deeper. Smaller particles in the 20 to 40 nm range migrate perifollicular over time instead.^71^ Where passive routes are insufficient, active platforms directly bypass the plug. Dissolving hyaluronic acid microneedles deposit cargo below the stratum corneum, and NIR-driven Zn-peroxide/MnO_2_ Janus nanomotors self-propel through *Cutibacterium acnes* biofilm after mechanical insertion.^10^ Collectively, these strategies demonstrate that the intrinsic polarity of metal oxide nanoparticles can be effectively managed through rational formulation design.^72^

## 3. Synthesis methods of metal oxide nanoparticles

**Fig. 3:**
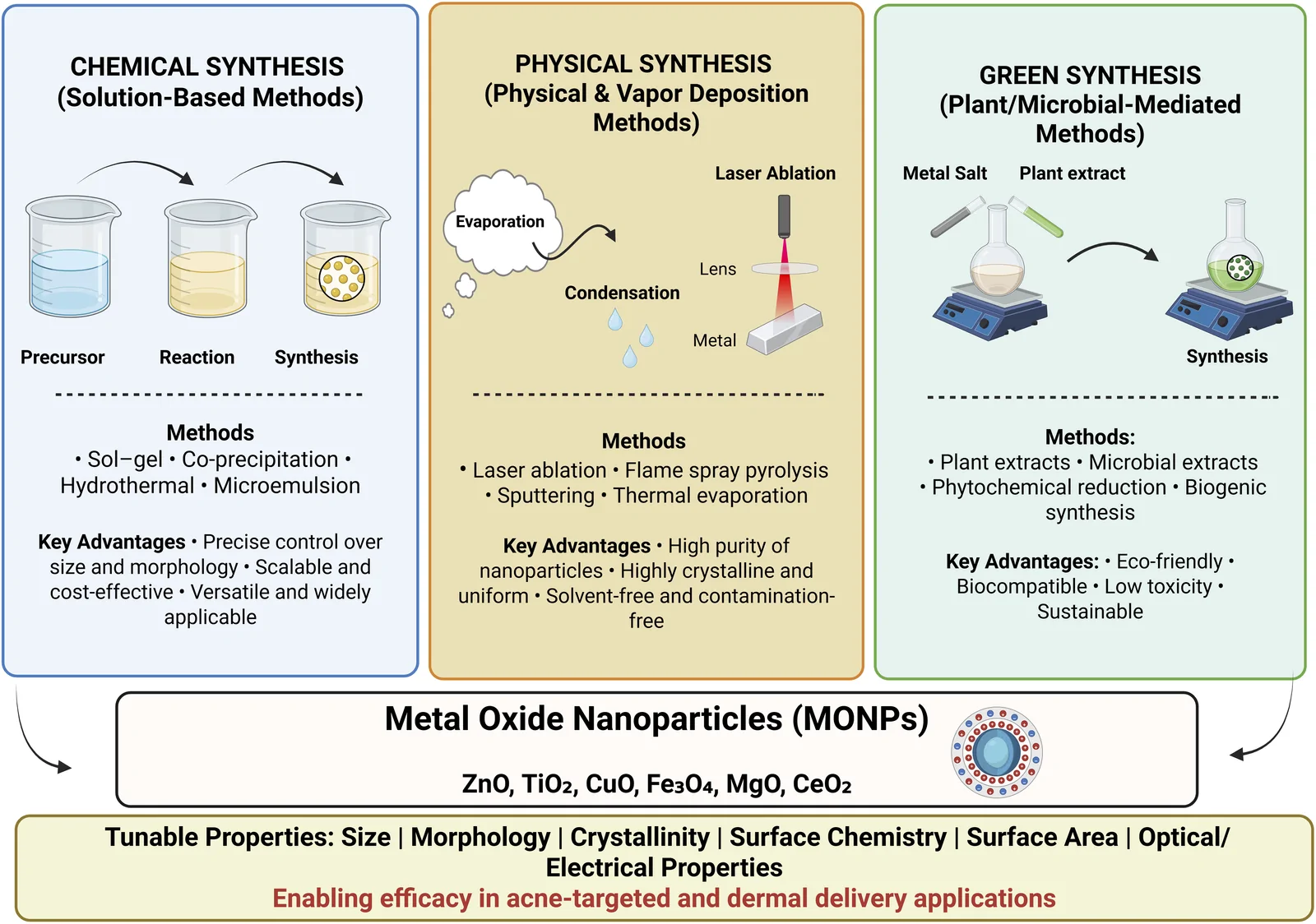
Overview of physical, chemical, and green synthesis methods used to prepare metal oxide nanoparticles. This image was created using BioRender software (https://www.biorender.com/).

The ability to tailor the particle size, morphology, crystallinity, and surface chemistry through controlled synthesis is fundamental to the design of MONPs for acne-targeted applications.^21^ The major synthesis approaches (physical, chemical, and biological) employed for the fabrication of MONPs are illustrated in Fig. 3. Each method offers distinct advantages and limitations with respect to particle quality, scalability, cost, and environmental impact.^26^

### 3.1 Physical methods

Physical methods, including laser ablation, sputtering, flame spray pyrolysis, and thermal evaporation, generally produce highly crystalline and impurity-free metal oxide nanostructures by vaporizing bulk metals or metal salts, followed by rapid quenching of the resulting vapor or plasma.^26,73^ Laser ablation in liquids can generate colloidal ZnO, TiO_2_, or Fe_3_O_4_ NPs with narrow size distributions (often 10–30 nm) and a well-defined wurtzite or spinel crystalline phase.^74–76^ The NPs exhibit excellent optical and magnetic properties for use in photocatalytic or photothermal acne therapy.^10^ Flame spray pyrolysis and aerosol-assisted methods also produce TiO_2_ and ZnO nanoparticles with controlled primary particle size and phase composition, which are widely used in commercial sunscreens and dermocosmetic formulations.^77^

However, these techniques typically require specialized equipment, significant energy input, and extensive post-processing steps, including milling, dispersion, and surface coating, to achieve stable aqueous suspensions for incorporation into topical gels or creams.^78^ In addition, control over particle aggregation may be limited, resulting in sub-micrometer hydrodynamic sizes that may favor follicular depot formation but could compromise deeper follicular penetration.^79^ Furthermore, because the particle surfaces are not inherently functionalized with organic capping agents, further modifications, such as silanization or polymer grafting, are often needed to improve biocompatibility and microbiome compatibility.^80^

### 3.2 Chemical methods

Wet chemical methods are the most common methods used to prepare MONPs with controllable morphology and surface chemistry.^81^ Sol–gel processes enable precise control over the hydrolysis and condensation kinetics of metal alkoxides or salts, facilitating the formation of oxides such as ZnO and TiO_2_.^82–85^ In these systems, parameters such as hydrolysis pH, solvent polarity, and aging time affect the network formation, porosity, and crystallite size. Nanorods, nanowires, or nanoplates can be highly crystalline and display a narrow size distribution when prepared by the hydrothermal/solvothermal method, which is usually carried out at 120–220 °C in sealed autoclaves.^8^ For example, wurtzite ZnO nanorods (length 200–400 nm; diameter 30–60 nm) expose polar crystal facets that enhance ROS generation and antibacterial activity.^86^

Co-precipitation and microemulsion methods are widely employed for the preparation of Fe_3_O_4_ and MgO nanoparticles. In these methods, metal ions are precipitated under alkaline conditions as hydroxides or related intermediates, followed by conversion to the desired oxide phase where required.^87,88^

Nucleation density and particle growth are highly sensitive to the reaction temperature and base addition rate, yielding particles ranging from ultrasmall superparamagnetic iron oxide nanoparticles (IONPs; 5 nm) to aggregates larger than 100 nm.^89^ The scalability of co-precipitation and the ability to incorporate dopants (*e.g.*, Ag, Cu, or Mn) into the oxide lattice to modulate ROS generation and broaden the antibacterial spectrum make this approach particularly attractive for acne therapy.^90^

### 3.3 Green chemistry principles for sustainable MONPs synthesis

Green synthesis approaches employ plant or microbial extracts to reduce, cap, and stabilize MONPs in aqueous media without the use of harsh reducing agents or organic solvents.^91^ Polyphenols, flavonoids, organic acids, and polysaccharides present in extracts of *Aloe vera*, neem, *Dendrobium species*, *Pluchea indica*, and other medicinal plants can induce the nucleation and growth of ZnO NPs.^92–94^ For example, the synthesis of ZnO NPs using *Aloe vera* produces NPs coated with ascorbic acid-rich phytochemicals, which not only imparts antioxidant and antibacterial characteristics to the NPs but also eliminates the need for additional surfactants.^95^

These bio-inspired pathway approaches closely align with the principles of green chemistry, including atom economy, the use of safer solvents, reduced energy consumption, and waste minimization, with most reactions being carried out in water at mild temperatures using non-toxic precursors.^96^ For plant-mediated ZnO and Ag/ZnO heterostructures, the elimination of organic ligands and simplified purification process significantly reduced the wastage of solvents and reagents, as evidenced by the results of life-cycle analyses, in comparison with multi-step sol–gel and combustion methods.^97–99^

Comparative studies indicate that green-synthesized ZnO and CuO NPs often exhibit comparable or superior antibacterial activity at lower MIC values despite their slightly broader size distributions, owing to the synergistic effects of the metal oxide core and bioactive capping molecules.^100^ However, problems exist in terms of batch-to-batch reproducibility, precise control of particle size, crystallinity, and standardization of extract composition from season to season and geographical sources.^26^ In the case of acne products, scalable processes with well-characterized extracts (*e.g.*, *Aloe vera*, neem) and robust process control will be essential to satisfy regulatory requirements for product quality while preserving the sustainability and safety benefits of green chemistry.^28,101,102^

## 4. Specific MONPs for acne therapy

Each class of metal oxides has a unique set of crystal structures, electronic properties, and ion-release kinetics, which influence their antibacterial spectrum, anti-inflammatory activity, and formulation compatibility.^90,103^ The following section summarizes the studies on individual oxide systems, including ZnO, TiO_2_, CuO, Fe_3_O_4_/γ-Fe_2_O_3_, MgO, CeO_2_, MnO_2_, and Al_2_O_3_, in terms of their mechanisms of action, quantitative efficacy data, formulation aspects, and safety, with a focus on structure–property relationships that can guide the rational design of materials for acne therapy.

### 4.1 Zinc oxide

Zinc is an essential trace element important for over 300 enzyme reactions, such as DNA repair, antioxidant protection, and innate and adaptive immunity.^104^ This role is closely related to acne pathophysiology because zinc controls the differentiation of keratinocytes, migration of neutrophils, and synthesis of IL-1β and IL-8 in the pilosebaceous unit, which influences comedogenesis and the intensity of the inflammatory response to *Cutibacterium acnes*.^104^ Thus, ZnO NPs can be considered as a ‘zinc reservoir’ in this context, which can release Zn^2+^ ions locally and simultaneously exhibit ROS mediated antibacterial, sebum regulating, and mild anti-inflammatory activity to connect the systemic trace element biology with localized acne therapy.^11^

Translating this biology into a delivery system requires an oxide whose physical form can be tuned to control the amount of Zn^2+^ released and its location. ZnO is most commonly used in the wurtzite crystal phase and can be prepared as nanospheres, nanorods, and nanoplatelets.^105^ It possesses a band gap of approximately 3.3 eV and surface properties that support UV absorption, ROS generation, and Zn^2+^ ion release.^106^ The particle sizes typically range from 20 to 70 nm, and green and hydrothermal synthesis methods are commonly employed to control the morphology and surface reactivity relevant to acne formulations.^107^

The acne-targeted activity of ZnO against *Cutibacterium acnes* and *Staphylococcus epidermidis* involves a combination of ROS-mediated oxidative damage, direct membrane interactions, and Zn^2+^-mediated enzyme inhibition.^108,109^ Smaller ZnO particles and those exposing high-energy facets generally generate higher levels of ROS and have lower MICs. Combinations of ZnO with organic acids (*e.g.*, citric acid) have demonstrated synergistic bactericidal activity against *Cutibacterium acnes in vitro*, especially with smaller particle size fractions. In addition, ZnO exhibits mild anti-inflammatory and sebum-modulating effects, partly through keratinocyte differentiation and 5α-reductase inhibition, making nanoscale formulations attractive as non-antibiotic acne therapeutics.^11^

A systematic evaluation of topical ZnO formulations revealed that hyaluronic acid-stabilized ZnO (HA-ZnO) reduced the burden of *Cutibacterium acnes* by 3.1 log_10_ on porcine skin within 24 h and achieved a 16-fold higher selective kill ratio over *Staphylococcus epidermidis* at 32 µg mL^−1^, highlighting its microbiome-sparing potential.^11^ Green-synthesized ZnO NPs produced using *Pluchea indica*, *Aloe vera*, and other plant extracts have shown MICs of approximately 250 µg mL^−1^ against *Cutibacterium acnes* and inhibition zones were 18–20 mm, which were comparable or superior to the activity of benzoyl peroxide at lower mass doses.^93,110^ Safety data on sunscreen and wound healing applications clearly demonstrate that ZnO NPs do not penetrate deeper than the stratum corneum and/or follicles, and that no intact ZnO NPs are found in the viable epidermis or in systemic organs under realistic exposure conditions.^42^ Some studies have reported that very high doses of ZnO may cause delayed wound closure or immunosuppression; therefore, it is necessary to optimize the dosage and surface coating in inflamed acne skin.^108^

### 4.2 Copper oxide (CuO, Cu_2_O)

Copper is another crucial trace element that functions as a cofactor for cuproenzymes, such as Cu/Zn-superoxide dismutase, cytochrome c oxidase, lysyl oxidase, and tyrosinase, aiding in mitochondrial energy generation, antioxidant protection, and collagen and elastin crosslinking in the skin.^111^ Alterations in systemic copper homeostasis have been linked to changes in oxidative stress and cytokine profiles in acne and other inflammatory skin disorders,^112,113^ suggesting that copper-dependent redox balance may influence sebum lipid peroxidation, neutrophil activation, and post-inflammatory tissue remodeling.^114^ At the nanoscale, precisely surface-engineered CuO/Cu_2_O systems may combine potent contact-killing activity against *Cutibacterium acnes* and co-colonizing staphylococci with localized modulation of extracellular matrix turnover and oxidative stress.^115,116^

However, this translational potential must be balanced against copper's relatively narrow therapeutic window and the risk of exacerbating inflammation if Cu^2+^ release is not adequately controlled in inflamed skin.^117^

**Fig. 4:**
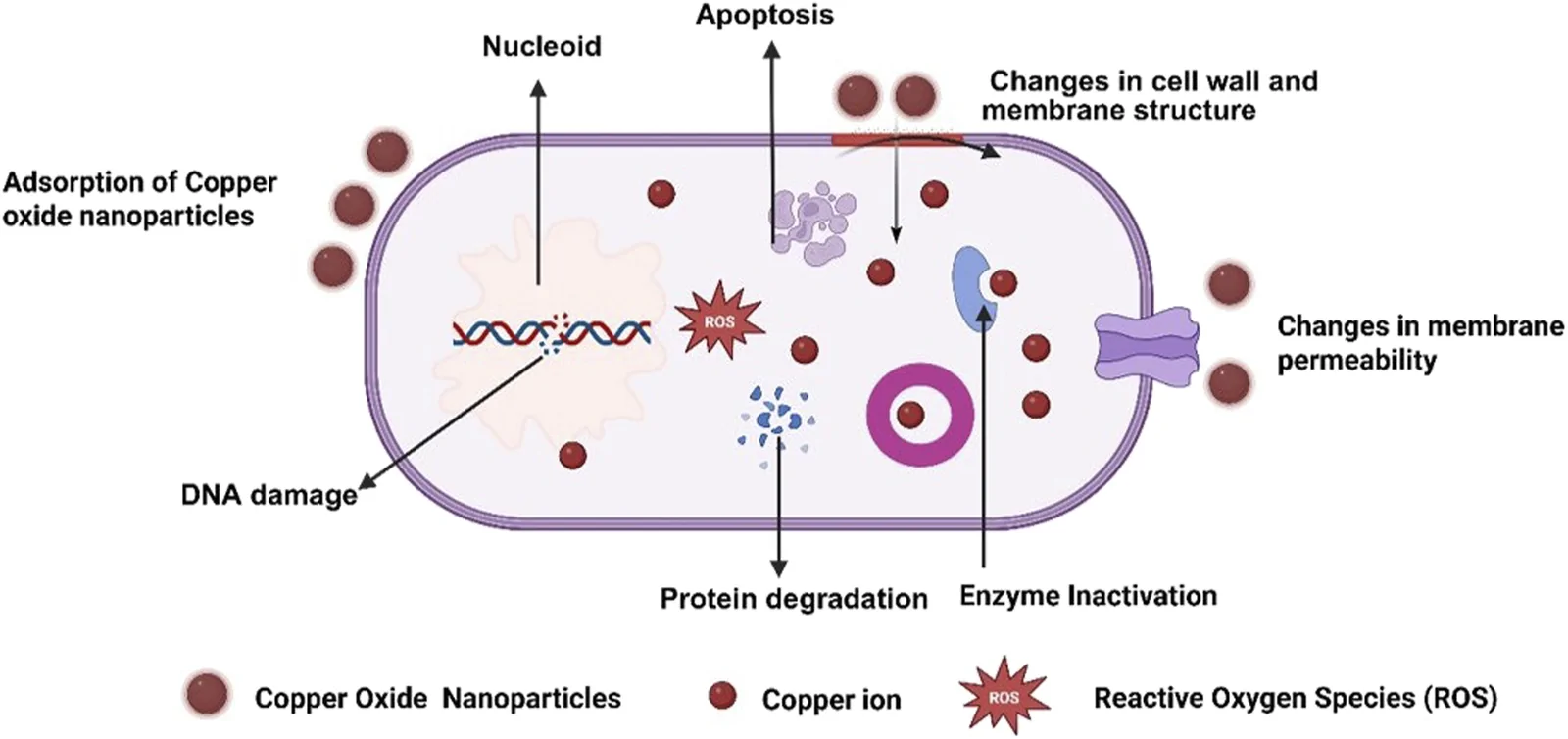
Illustration of the antibacterial mechanisms of copper oxide nanoparticle. This image was created using BioRender software (https://www.biorender.com/).

CuO and Cu_2_O NPs possess p-type semiconducting properties and rich redox chemistry (Cu^2+^/Cu^+^ cycling),^118^ which underpins their strong antibacterial and antibiofilm activities.^14^ The particle size of chemically or green-synthesized CuO NPs ranged from 10.7 to 30.9 nm. They exhibit high ROS generation and copper ion release, enabling effective killing of Gram-positive bacteria, including *Cutibacterium acnes* and *Staphylococcus aureus*, at relatively low concentrations.^119^ A positive surface charge increases the electrostatic attraction to bacterial membranes, promoting membrane disruption, leakage of cytosolic contents, and metabolic collapse (Fig. 4).^120^

In acne-relevant contexts, chitosan films and hydrogels incorporating green-synthesized CuO and Ag NPs have demonstrated strong antibacterial and antibiofilm activity while maintaining acceptable mammalian cell compatibility, with MICs in the low to mid tens of µg mL^−1^ against clinically relevant bacteria. These composite systems also substantially inhibit biofilm formation.^33^ Mechanochemically generated ultrasmall CuO NPs also have a size-dependent antibacterial effect, with 7 and 14 nm particles inducing rapid log_10_ reductions in *Staphylococcus aureus* at sub-mg mL^−1^ doses, which can be used as prototypes for low-dose acne dressings.^121^ CuO NPs can also be combined with iron oxide-based nanocomposites, where the synergistic effect of pH-responsive Cu^2+^ release and the magnetic or photothermal effect is exploited to eradicate biofilms and inhibit inflammatory signalling in an infection model, suggesting the possibility of CuO NPs as composite platforms for acne therapy.^34^

Surface chemistry and synthesis pathways have a significant impact on safety.^122^ Bare CuO NPs are relatively cytotoxic because of the fast release of Cu^2+^ and high ROS production, which results in mitochondrial damage and pro-inflammatory effects in keratinocytes at high doses, as shown in toxicological studies.^123^ However, CuO NPs synthesized with plant or fungal biomolecules as capping agents or polymer-modified CuO NPs (such as polydopamine-coated CuO NPs) exhibit reduced ion leaching and cytotoxicity while demonstrating improved dispersion stability and strong antibacterial activity.^124^

### 4.3 Titanium dioxide

Titanium, unlike zinc and copper, is not an essential trace metal in humans and is generally regarded as a biologically inert metal. Its oxide (TiO_2_) is extensively used in orthopaedic implants and sunscreens because of its remarkable biocompatibility and resistance to corrosion, rather than any inherent physiological role.^125^ In acne vulgaris, TiO_2_ can be viewed as an external, follicle-targeted UV filter and photocatalyst that supports disease management by reducing UV-induced oxidative stress, post-inflammatory hyperpigmentation, and treatment-related photosensitivity while, under controlled irradiation, generating sufficient ROS to inactivate *Cutibacterium acnes* without significant penetration into viable skin layers.^126^

Rutile or surface-coated anatase-rutile TiO_2_ NPs with primary sizes ranging from 20–100 nm may aggregate into larger secondary particles in dermatological formulations while still providing broad-spectrum UV protection. This property is particularly relevant for acne management, as it helps reduce UV-induced inflammation and post-inflammatory hyperpigmentation.^127^

TiO_2_ NPs exhibit antibacterial activity against *Staphylococcus aureus* and other skin pathogens through UV-activated ROS generation (including hydroxyl radicals, superoxide anions, and hydrogen peroxide), direct membrane damage, and biofilm disruption.^128^

Mixed-phase anatase/rutile systems are often more effective than single-phase materials.^129^ TiO_2_ NPs have also been found to act synergistically with conventional antibiotics, lowering the effective antibiotic doses against multidrug-resistant strains. Such combinations may be useful in acne treatment regimens aimed at minimizing the reliance on systemic antibiotics.^130^

Dermal penetration studies in human volunteers and *ex vivo* skin models have consistently shown that TiO_2_ nanoparticles remain confined to the stratum corneum and hair follicles, with negligible penetration of intact particles into the viable epidermis or dermis, even after repeated application.^131^ In addition, green-synthesized TiO_2_ prepared using plant extracts has shown broad-spectrum antibacterial activity and may have improved biocompatibility compared with chemically synthesized TiO_2_, making it a promising sustainable candidate for use in acne cleansers or leave-on formulations to reduce microbial and sebum oxidation.^132^ Formulations targeting acne may exploit the dual role of TiO_2_ as both a UV filter and photocatalyst, provided that ROS generation within viable skin layers is carefully controlled through appropriate surface coatings and photostabilizing matrices.^18^

### 4.4 Iron oxide

Iron is essential for oxygen transport, mitochondrial respiration, and the function of various redox enzymes.^133^ In the context of acne, iron-dependent ROS generation within sebum and surrounding tissues may exacerbate comedogenesis and inflammation, although a direct relationship between systemic iron deficiency or excess and acne severity has not been clearly established.^134^ Iron oxide NPs (Fe_3_O_4_/γ-Fe_2_O_3_) harness iron's inherent redox properties into a controllable nanozyme platform, in which Fenton-like reactions and photothermal conversion can be localized within *Cutibacterium acnes* biofilms and inflamed follicles upon exposure to near-infrared (NIR) light and/or H_2_O_2_. This enables potent antibacterial and antibiofilm effects while avoiding the systemic iron imbalances associated with conventional iron supplementation.^34^

The realization of this redox potential as a therapeutic tool depends on the physical form of iron oxide, which governs how readily it behaves as a catalytic nanozyme rather than an inert depot. Iron oxide nanoparticles (IONPs), particularly magnetite (Fe_3_O_4_) and maghemite (γ-Fe_2_O_3_), exhibit superparamagnetic behaviour at sizes below approximately 20–30 nm and display enzyme-like (nanozyme) catalytic activity.^135^ Their peroxidase and catalase-like activities enable Fenton-type reactions, in which H_2_O_2_ is converted to hydroxyl radicals and other ROS under mildly acidic conditions, a property amenable to the eradication of bacteria within biofilms associated with acne lesions.^136^

Fe_3_O_4_ NPs with high photothermal conversion efficiency and peroxidase activity have been reported to achieve near-complete killing of *Escherichia coli* and *Staphylococcus aureus* under near-infrared (NIR) irradiation in the presence of H_2_O_2_, while simultaneously accelerating healing in infected wound models.^137^ Translating this concept to acne, mesoporous Fe_3_O_4_ based nanocomposites designed for synergistic photothermal/Fenton therapy have been shown to generate potent ROS within bacterial biofilms under NIR irradiation, thereby reducing bacterial load and inflammatory markers.^34^ Doping or compositing Fe_3_O_4_ nanoparticles with other metals (*e.g.*, Cu and Ag) can further enhance their antibacterial potency and broaden their spectrum of activity.^138^

IONPs are among the most clinically advanced inorganic NPs, with established applications as MRI contrast agents and iron-replacement therapies, and they generally exhibit favorable safety profiles when appropriately surface-modified.^139^ Dermal toxicity tests suggest that surface-modified Fe_3_O_4_ NPs are generally well tolerated at therapeutic doses but may cause oxidative stress at high local doses owing to dose–dependent ROS generation.^140^ Chitosan or polymer-coated Fe_3_O_4_ systems that can accumulate in pilosebaceous units and are activated by mild NIR irradiation are a controllable, non-antibiotic way to treat acne, especially recalcitrant nodular lesions.^141^

### 4.5 Magnesium oxide

Magnesium is the most abundant intracellular divalent cation and a cofactor for enzymes in ATP-dependent metabolism, nucleic acid synthesis, and signalling. It is also involved in the maintenance of epidermal barrier homeostasis, hydration and pH balance.^142^ In patients with acne, lower serum magnesium levels were found in those with more severe disease and in atopic dermatitis and xerosis models, there is evidence that magnesium-rich salts enhance barrier function, stratum corneum hydration, and decrease cutaneous inflammation. These observations suggest a broader role for magnesium in modulating inflammatory responses in the skin.^143^

By providing Mg^2+^ in a well-dispersed, large-surface-area form, MgO NPs can preferentially target acne-related Gram-positive bacteria while also aiding in local barrier repair and reducing pro-inflammatory signalling in the pilosebaceous unit, as long as the particle dosage and surface basicity are precisely adjusted to prevent irritation of sensitive acne-prone skin.^144,145^

Delivering magnesium locally and in a controllable form is where MgO NPs become useful: their alkaline surface chemistry and intrinsic biocompatibility render them attractive for dermatological applications. Their antibacterial activity is primarily attributed to ROS generation, surface basicity, and direct membrane interactions.^16^ The MICs for quasi-spherical MgO NPs (25–60 nm) synthesized by co-precipitation or green-synthesis methods are in the range of 0.5 to 1 mg mL^−1^ against *Staphylococcus aureus*, *Staphylococcus epidermidis* and other Gram-positive bacteria, while the activity is comparatively lower against Gram-negative bacteria.^146^ This preferential activity against Gram-positive bacteria is consistent with the pathophysiology of acne, in which *Cutibacterium acnes* and commensal staphylococci are the predominant microorganisms.^147^

In a study by Yusop *et al.*, MgO NPs were incorporated into coated fabrics, resulting in a significant decrease in the number of viable *Cutibacterium acnes* on cotton, polyester, and wool fabrics, providing proof of concept for the development of anti-acne textiles and wound dressings.^148^ In addition, the combined effect of photosynthesized MgO NPs and essential oils results in synergistic antibacterial activity, with a lower MIC value and better membrane disruption compared to each individual factor, which could be due to ROS generation and lipophilic disruption of bacterial envelopes.^149^ MgO NPs have also demonstrated anti-inflammatory and antioxidant effects in bioinspired formulations, including reductions in free radical levels and modulation of inflammatory pathways in preclinical models, which may be beneficial for inflamed acne lesions.^150^

Available *in vitro* toxicity data suggest that MgO NPs possess relatively wide safety margins, with overt toxicity generally not observed until concentrations reach several hundred µg mL^−1^.^151^ In the field of acne formulations, MgO can be used as a co-active ingredient in gels or films, where both pH control and mild sebum adsorption are desired.^152^

### 4.6 Cerium oxide

Although cerium has no known physiological role in humans, CeO_2_ NPs can mimic the functions of superoxide dismutase and catalase through their ability to alternate between Ce^3+^ and Ce^4+^ oxidation states and dynamically control ROS in redox-active microenvironments.^153^ This characteristic is particularly relevant to acne lesions, where elevated oxidative stress, lipid peroxidation of sebum, and activation of redox-sensitive pathways (such as NF-κB and the NLRP3 inflammasome) maintain perifollicular inflammation and tissue injury.^134^

When suitably surface-modified, CeO_2_ nanoparticles can enhance endogenous antioxidant mechanisms within pilosebaceous units, while concurrently reducing oxidative and inflammatory processes and exhibiting mild antibacterial properties, establishing CeO_2_ as a unique “redox-modulating” nano-oxide for the treatment of inflammatory and post-inflammatory acne.^154^

The defining feature of CeO_2_ NPs is their capability to switch between the oxidation states of Ce^3+^ and Ce^4+^, which enables them to possess high superoxide dismutase and catalase mimicking activity, as well as regenerative antioxidant behavior.^155^ This redox flexibility enables CeO_2_ to scavenge ROS under conditions of oxidative stress and contributes to localized antibacterial oxidative activity under certain circumstances. This dual behavior is highly relevant to inflammatory acne lesions, where both oxidative stress and bacterial colonization coexist.^17^

Numerous studies have demonstrated that CeO_2_ NPs mitigate oxidative stress, suppress inflammatory pathways such as the NLRP3 inflammasome and NF-κB, and induce macrophage polarization towards pro-healing phenotypes, thereby enhancing tissue repair in wound healing, diabetic ulcer, and psoriasis models.^156^ Cyclodextrin-modified CeO_2_ NPs have also shown strong anti-inflammatory activity in psoriasis models by significantly reducing disease severity scores, lipid peroxidation, and pro-inflammatory cytokine expression.^157^ A study by Nosrati *et al.* reported that CeO_2_ topical formulations exhibited enhanced antibacterial activity and improved resolution of inflammatory lesions, although detailed clinical data remain limited.^158^

For acne therapy, nanoceria may function as a dual-action agent by attenuating oxidative stress and inflammation in pilosebaceous units while exerting surface-level antibacterial effects through membrane interactions and modest ROS generation.^159^ Coating strategies involving polymers or biomolecule coatings reduce aggregation, improve dispersion in hydrogels, and modulate redox activity to avoid excessive ROS in viable skin.^160^ Toxicological assessments highlight dose–dependent effects and emphasize the need to balance antioxidant and pro-oxidant activities. Appropriately coated ultrasmall CeO_2_ NPs have demonstrated efficient clearance and acceptable safety in systemic models, suggesting that well-designed topical systems may achieve a satisfactory safety margin.^161^

### 4.7 Manganese oxides

Manganese is an important trace element and a cofactor for mitochondrial manganese superoxide dismutase (MnSOD), as well as several enzymes involved in antioxidant defence, collagen synthesis, connective tissue formation, energy metabolism and skin wound healing.^162^ Sufficient Mn-dependent MnSOD activity is essential to restrain mitochondrial ROS and apoptosis in UV radiation-and inflammatory stress-induced keratinocytes and fibroblasts, which can otherwise contribute to acne-related erythema, matrix degradation, and scarring.^163^ MnO_2_-based nanostructures used in acne microneedle and nanomotor platforms can therefore be viewed not only as catalytic oxygen generators within hypoxic *Cutibacterium acnes* biofilms but also as exogenous modulators of Mn-dependent redox pathways in the surrounding tissue. However, careful control of dose, activation conditions, and degradation is required to avoid tipping the balance towards excessive oxidative stress.^10^

Manganese oxides are of interest in acne therapy, primarily as catalytic and oxygen-generating components in composite nanoplatforms.^10^ MnO_2_ nanosheets and shell architectures can decompose endogenous H_2_O_2_ in acidic microenvironments to produce molecular oxygen, thereby alleviating local hypoxia while simultaneously generating hydroxyl radicals in conjunction with Fenton-type catalysts.^164^

One of the most innovative examples is a NIR-driven nanomotor-based microneedle system for the active treatment of acne. In this approach, zinc peroxide NPs were asymmetrically modified with polydopamine, followed by *in situ* MnO_2_ growth to form Janus Z@P-M nanomotors, which were subsequently loaded into dissolvable HA microneedles. Upon topical application and 808 nm laser irradiation, the microneedles delivered Z@P-M nanomotors into the dermis, where zinc peroxide slowly released H_2_O_2_ in the acidic *Cutibacterium acnes* biofilm microenvironment. MnO_2_ then catalyzes the conversion of H_2_O_2_ to O_2_, alleviating hypoxia and promoting ROS-mediated bacterial killing. This polydopamine layer creates a photothermal gradient that induces the active penetration of nanomotors into dense biofilms and enhances the distribution of nanomotors within lesions. In murine acne models, this system has been shown to significantly decrease *Cutibacterium acnes* biofilms, suppress inflammatory cytokines, and restore immune homeostasis, exemplifying the complex design of this system comprising catalytic MnO_2_ chemistry, photothermal actuation, and active transport.^10^

### 4.8 Aluminium oxide (Al_2_O_3_) and other emerging oxides

In contrast to zinc, copper, iron, magnesium, and manganese, aluminium is not regarded as an essential element for human physiology, and its compounds are generally considered pharmacologically inert at typical dermal exposure levels, with safety concerns arising mainly from chronic systemic accumulation rather than from any defined deficiency state.^165^ When incorporated at low levels into composite platforms, Al_2_O_3_ may contribute to the performance of acne-targeted formulations^166^ while also improving the textural or rheological properties of topical systems for oily, acne-prone skin, provided that total aluminium exposure remains within established cosmetic safety margins and that long-term dermal accumulation is carefully evaluated.^167^

Although Al_2_O_3_ nanoparticles have been less extensively investigated for acne therapy, they have been reported to exhibit antibacterial activity through reactive oxygen species (ROS) generation and electrostatic interactions with bacterial membranes.^168^ Bacteriostatic effects have been reported against a variety of Gram-positive and Gram-negative bacteria, although relatively high concentrations of NPs are often required.^169^ Cytotoxicity towards mammalian cells is generally low at comparable concentrations, particularly for the α-phase of Al_2_O_3_, which exhibits lower ROS generation, suggesting a potentially favourable safety profile.^170^ Although direct evidence against *Cutibacterium acnes* remains limited, Al_2_O_3_ nanoparticles may serve as adjuvant antibacterial agents or carriers in composite acne formulations, where their mechanical hardness and chemical stability can be advantageously exploited.^169,171^

Other oxides, such as silver oxide (Ag_2_O), cobalt oxides (Co_3_O_4_), and zirconia (ZrO_2_), have demonstrated excellent anti-inflammatory activity in wound and implant applications and have been suggested as possible candidates for acne-relevant formulations, particularly if they are green-synthesized to enhance their biocompatibility. However, their translation to acne therapy requires careful toxicological optimization and dedicated studies evaluating their effects on *Cutibacterium acnes* and the broader skin microbiome. A brief overview of MONPs investigated for antibacterial action, along with their mechanisms of action and therapeutic outcomes, is presented in Table 2.

**Table 2:** Overview of metal oxide nanoparticles with antibacterial action

Metal oxide nanoparticles	Primary mechanism(s)	Description	References
Zinc oxide (ZnO-NPs)	ROS generation and Zn^2+^ release; membrane disruption; suppression of pro-inflammatory signalling (IFN-γ/TNF-α-driven iNOS)	A preclinical and clinical study of hyaluronic acid-stabilized ZnO NPs showed a 16-fold higher bactericidal ratio against *Cutibacterium acnes* than against *Staphylococcus epidermidis*, supporting a microbiome-sparing selectivity profile	172
Iron oxide (Fe_3_O_4_ NPs)	Fenton/Fenton-like hydroxyl radical (·OH) generation; photothermal membrane disruption	A PMS-modified mesoporous iron oxide nanocomposite co-loaded with CuO and quercetin releases Fe^2+^/Cu^+^ under the acidic acne microenvironment (pH ≈ 5), generating ·OH that, combined with NIR-photothermal membrane disruption, achieves complete biofilm eradication	34
Manganese oxide (MnO_2_-NPs)	Catalytic O_2_ generation and hypoxia alleviation; NIR-driven photothermal membrane disruption	Asymmetric ZnO_2_@polydopamine-MnO_2_ nanomotors delivered *via* microneedle bypass the stratum corneum; a NIR-induced thermal gradient enhances diffusion and biofilm penetration, enabling combined oxygenation-based and photothermal anti-*Cutibacterium acnes* therapy	10
Copper oxide nanoparticles (CuO NPs)	Sustained Cu^2+^ release; high-efficiency ROS generation	Green-synthesized CuO-NPs incorporated into chitosan films (CuONPs@CHI) showed selective anti-*Cutibacterium acnes* activity (MIC = 62 µg mL^−1^; MBC = 125 µg mL^−1^), supporting their use as a targeted anti-acne biomaterial	33
Magnesium oxide nanoparticles (MgO-NPs)	ROS generation; Ca^2+^ dysregulation; quorum sensing interference; membrane disruption	MgO-NPs act through multiple, non-mutually exclusive mechanisms; oxidative damage, disruption of bacterial Ca^2+^ handling, interference with quorum sensing, and direct membrane damage producing broad-spectrum antibacterial activity	16
Aluminium oxide (Al_2_O_3_-NPs)	Electrostatic membrane adhesion with positive zeta potential; Al^3+^ induced ROS and genotoxicity	Positively charged Al_2_O_3_ NPs adsorb electrostatically to bacterial envelopes. They are more effective against gram-negative species. Released Al^3+^ promotes membrane lipid peroxidation and ROS-mediated DNA damage; strand breaks, base modification, DNA-protein crosslinks. Their effects are dependent on dose and morphology	169

### 4.9 Comparative evaluation of oxide platforms

Although these oxides share general antibacterial properties, treating them as a uniform class oversimplifies their clinical utility. Based on the strength of acne-specific evidence and their potential for clinical translation, the major metal oxide nanoparticle platforms are discussed in this section.

Zinc oxide has the strongest and most acne-relevant evidence. The mechanism is well defined: pH-dependent Zn^2+^ release combined with mild ROS generation drives the reported activity against *Cutibacterium acnes*. This is further supported by early human evidence for HA-stabilized nanogels and regulatory approval as a sunscreen UV filter.^11,42^ However, its principal constraints are dose–dependent effects on keratinocyte function at high loadings and the need to tune coating and release for inflamed skin.^108^ Iron oxide represents the next most promising oxide platform. Its established clinical use as an MRI contrast agent and in iron-replacement therapy provides a favorable translational precedent. It also offers a controllable NIR/Fenton mechanism. However, acne data remain preclinical and depend on external activation.^34,139^

Copper oxide is mechanistically potent against *Cutibacterium acnes* at low concentrations. However, its narrow therapeutic window and rapid Cu^2+^ release, combined with documented keratinocyte cytotoxicity and genotoxicity, make uncoated formulations difficult to justify for daily facial use. Capped or polymer-modified variants can be a realistic route forward.^33,123,124^ Titanium dioxide works best as a follicle-targeted UV filter and controlled photocatalyst rather than as a primary antibacterial agent. Extensive skin safety data strongly supports this role.^127,131^ Cerium oxide represents another promising oxide platform. It is a promising anti-inflammatory adjuvant despite its modest direct anti *Cutibacterium acnes* activity. Furthermore, most supporting data are limited to wound healing and psoriasis models.^17,156,157^ Magnesium oxide is well tolerated and preferentially kills Gram-positive acne bacteria. However, the evidence for this relies on textiles and general antibacterial assays instead of clinical acne trials.^146,148^ Manganese oxide serves almost exclusively as a catalytic oxygen-generating component within composite microneedle platforms. Because it is rarely tested alone, its independent performance remains unproven apart from the delivery system.^10^

Aluminium oxide currently has a weak evidence base as it is only bacteriostatic at high concentrations and lacks direct *Cutibacterium acnes* evidence. Additionally, chronic accumulation concerns confine it strictly to an adjuvant or rheological role.^165,168,169^ The CuO, Fe_3_O_4_ and MnO_2_ systems are better suited for the targeted and activation-dependent treatment of recalcitrant lesions. The standardized efficacy data underlying this appraisal are presented in Table 3.

**Table 3:** Standardised summary of antibacterial efficacy reported for the oxide systems

Oxide	Particle size (nm)	Synthesis	Test organism	Antibacterial metric	Acne formulation
ZnO^11,93,110^	20–50 nm	Green/hydrothermal	*Cutibacterium acnes*; 2 clinical isolates and NCTC 747 reference strain	MIC = 250 µg mL^−1^ (broth microdilution); sub-MIC biofilm inhibition active at 15–62.5 µg mL^−1^ (strain-dependent)	Hyaluronic acid-coated ZnO nanoparticles
					Gel-based formulations
					Nanofibre carrier systems
CuO^33^	9.11 ± 6.72 nm by HRTEM	Green/chemical	*Cutibacterium acnes* (reference strain)	MIC = 62 µg mL^−1^; MBC = 125 µg mL^−1^ (broth microdilution)	Chitosan film demonstrated selective bactericidal efficacy against *Cutibacterium acnes* over *Escherichia coli* and *Staphylococcus aureus* in a head-to-head study, supporting a targeted rather than broad-spectrum therapeutic design
CuO (ultrasmall)^121^	A narrow size distribution was observed, with median particle sizes of 7 and 14 nm for CuCl_2_·2H_2_O and CuSO_4_·5H_2_O precursors, respectively	Mechanochemical	*Staphylococcus aureus* (model)	Low MIC values at sub-mg mL^−1^ doses against *Staphylococcus aureus* and *Escherichia coli*	Prototype for low-dose acne dressing
TiO_2_ (ref. 173 and 174)	DLS measurements showed an average particle size of 74.8 ± 0.649 nm	Sol–gel/green	*Staphylococcus aureus*, *Escherichia coli* (Gram-positive/-negative surrogates; no *Cutibacterium acnes* data available)	Log reduction = 3.34 log (*Staphylococcus aureus*) and 3.7 log (*Escherichia coli*) at 2% w/v under photoactivating light, from a 5 log CFU cm^−2^ inoculum (18 h contact)	Efficacy is photocatalysis-dependent and shown only against surrogate organisms; no *Cutibacterium acnes*-specific data are available
	TEM studies revealed diameter to be in the range of 10–30 nm				
Fe_3_O_4_ & Fe_2_O_3_ (ref. 175)	238.5 ± 9.67 nm (hydrodynamic size)	Co-precipitation	*Cutibacterium acnes* ATCC 6919, *Staphylococcus aureus* ATCC 29213	MIC = 0.25–0.5 mg mL^−1^ (250–500 µg mL^−1^) across three IONP molarities; formulated pickering emulsion achieved >97% reduction (% *R*) against both organisms within 2 h contact	One of the few oxides with paired MIC *and* finished-formulation reduction data against *Cutibacterium acnes* in a dual UV-protective cosmeceutical context
MgO^16^	23 ± 5 nm by SEM analysis	Co-precip./green	*Staphylococcus aureus*, *Staphylococcus epidermidis* (no *Cutibacterium acnes* data available)	MIC = 0.5–0.7 mg mL^−1^ (500–700 µg mL^−1^); MBC_90_ = 0.7 mg mL^−1^	Effective concentration is roughly one order of magnitude higher than ZnO or CuO, consistent with an adjuvant/pH-buffering role rather than a primary antibacterial agent; *Cutibacterium acnes* -specific testing remains an evidence gap
CeO_2_ (ref. 176)	5–20 nm	Wet-chemical/green	*Staphylococcus aureus* among a gram-positive/-negative panel (no *Cutibacterium acnes* data available)	MIC = 10 mg mL^−1^; MBC = 10 mg mL^−1^ for susceptible strains, with *Staphylococcus aureus* among the less-sensitive organisms tested	Weaker, dose–dependent, redox–switchable activity positions CeO_2_ as an anti-inflammatory adjunct rather than a primary antibacterial oxide; *Cutibacterium acnes* -specific testing is absent
MnO_2_ (ref. 10, 177 and 178)	Crystallite sizes of less than 10 nm	*In situ* growth	*Cutibacterium acnes*, murine acne model (biofilm-associated)	Bactericidal/anti-inflammatory efficacy reported only for the composite nanomotor-microneedle system *via* biofilm-oxygenation and NIR-photothermal action; no standardized MIC or log-reduction value exists for MnO_2_ in isolation	Z@P-M nanomotor in HA microneedle
Al_2_O_3_ (ref. 168 and 169)	Average crystallite size (XRD) = 4.6 nm	Chemical	*Escherichia coli*, *Klebsiella pneumoniae*, *Pseudomonas aeruginosa*, *Staphylococcus aureus* (no *Cutibacterium acnes* data available)	Inhibition zones 9–14 mm; MIC = 4–16 mg mL^−1^ (4000–16 000 µg mL^−1^), one to two orders of magnitude higher than ZnO	Authors concluded that Al_2_O_3_ alone did not show promising antibacterial results, supporting its classification as an adjuvant/rheology modifier rather than an active antibacterial component
	Average particle size (TEM) = 4.1 nm				

### 4.10 *Cutibacterium acnes*-directed activity and microbiome sparing

Membrane disruption and ROS production are general bactericidal mechanisms. On their own, they cannot distinguish *Cutibacterium acnes* from the non-pathogenic *Staphylococcus epidermidis*.^33,63^ These mechanisms operate at the level of the formulation and microenvironment rather than at the molecular level. The anatomical localization of cutaneous microbes necessitates targeted drug delivery.^63,179^ While *Cutibacterium acnes* resides deep within the hypoxic follicular microenvironment, *Staphylococcus epidermidis* occupies the superficial skin layers.^63,180^ Formulations engineered for follicular retention concentrate the active drug directly at the primary site of infection, limiting off-target exposure to the protective surface microbiome.^179^

Microenvironment-gated release adds a second layer of therapeutic specificity. The biofilm niche within an inflamed follicle is locally acidic, hypoxic, and enriched in H_2_O_2_.^34,181^ This environment accelerates ion release and Fenton-type ROS generation from oxides such as ZnO, CuO, and Fe_3_O_4_, preferentially inside the niche.^34,172^ The dose and release kinetics are other important factors. Sustained low-level ion release from a coated depot can suppress a susceptible follicular population while keeping peak concentrations below those that perturb the wider commensal community.^33,172^

Chitosan films functionalized with green-synthesized CuO NPs demonstrated significant antibacterial selectivity towards *Cutibacterium acnes*.^33^ Hyaluronic acid-stabilized ZnO demonstrated comparable *in vitro* selectivity, achieving a higher bactericidal ratio against *Cutibacterium acnes* than against non-pathogenic *Staphylococcus epidermidis*.^11,172^ Rather than demonstrating absolute species selectivity, these differential survival rates suggest a narrow but clinically exploitable therapeutic window.^33,172^ However, translating these findings to clinical applications requires caution. Robust microbiome-resolved investigations utilizing 16S rRNA or shotgun metagenomic sequencing before and after treatment remain imperative before any metal oxide formulation can be definitively classified as microbiome-sparing in human subjects.^11,63,180^

### 4.11 Trigger-dependent redox behaviour: reconciling antioxidant and ROS-generating activity

These two behaviors largely belong to different oxides. Photocatalytic ROS generation is a property of wide-bandgap semiconductors, such as TiO_2_ and ZnO, under illumination.^77,182^ In contrast, ROS scavenging is characteristic of CeO_2_ through its reversible Ce^3+^/Ce^4+^ couple.^183^

In some cases, a single oxide can act in both directions of the reaction. When it does, the direction is set by a defined trigger rather than occurring simultaneously. For TiO_2_ and ZnO, the trigger is photonic. Only photons at or above the bandgap energy excite electron–hole pairs that drive interfacial ROS production.^77,182^ This threshold is approximately 390 nm for anatase TiO_2_ and 375 nm for ZnO.^182^ Under ambient visible light or in the dark, these oxides are comparatively inert and behave as UV filters rather than oxidants.^77,182^

For CeO_2_, the trigger is the local redox and pH environment. At physiological to mildly alkaline pH, the high density of surface-bound Ce^3+^ ions enables nanoceria to mediate reactive oxygen species (ROS) scavenging *via* intrinsic superoxide dismutase and catalase-like activities. Upon introduction to an acidic and oxidatively stressed microenvironment, the underlying Ce^3+^/Ce^4+^ redox equilibrium shifts to effectively transform the NPs into pro-oxidant agents.^183^ Iron oxides operate under a comparable pH-responsive framework. In highly acidic local conditions, their intrinsic Fenton chemistry acts *via* a peroxidase-like mechanism, rapidly converting H_2_O_2_ into highly reactive hydroxyl radicals.^184^ This activity is switched on by the acidic, peroxide-containing follicular biofilm and NIR heating and remains minimal outside these conditions.^10,34^

Each behaviour should be interpreted in the context of its specific trigger. The trigger may be a wavelength, pH range, or H_2_O_2_ level. This removes the contradiction, and for acne, it also defines the conditions under which each oxide should be used. TiO_2_ and ZnO function as controlled photoactive systems.^77,182^ CeO_2_ primarily functions as an antioxidant under physiological conditions.^183^ Fe_3_O_4_ and MnO_2_ function as microenvironment-gated oxidants inside the biofilm.^10,34,184^

## 5. Formulation strategies and delivery systems

To enhance follicular targeting, control release, and enable combination with existing therapeutic agents, acne-targeted MONP formulations employ hydrogels, nanogels, lipid- and polymer-based carriers, microneedles, and smart nanomotor systems.^10,21^ Chitosan, alginate, gellan gum, hyaluronic acid (HA), and cellulose-derived hydrogels can be used to incorporate ZnO and MgO nanoparticles and deliver them in close proximity to the pilosebaceous units.^185^ Preclinical and early clinical studies have shown that ZnO loaded hydrogels and nanogels exhibit prolonged zinc release, enhanced viscosity and spreading, and substantial reductions in *Cutibacterium acnes* burden and inflammatory lesions.^186^ Green-synthesized TiO_2_ or CeO_2_ NPs incorporated into gellan or polysaccharide hydrogels as wound dressings have demonstrated good antibacterial and antioxidant activities, which can be easily translated into acne patches and spot treatments.^187^

Nanomotors and stimulus-responsive systems add an active dimension to drug delivery.^188,189^ The NIR-driven Z@P-M Janus nanomotor microneedle patch delivers self-oxygenating Zn-peroxide/MnO_2_ nanomotors to the dermis, where NIR-induced self-thermophoresis promotes deep biofilm penetration, and catalytic H_2_O_2_ decomposition alleviates hypoxia while enhancing ROS-mediated killing.^10^ These micromotile systems illustrate how MONPs can be implemented in smart platforms that are responsive to light and microenvironmental stimuli, such as pH and H_2_O_2_ concentration.^10,190^

To improve follicular targeting and control the release kinetics of MONPs, these nanoparticles can be encapsulated within or surface-decorated with liposomes, solid lipid nanoparticles (SLNs) and polymeric nanoparticle carriers.^191^ In the case of hair follicle delivery, studies have shown that lipid-based particles in the 200–400 nm range can efficiently accumulate in the follicles and that their surface charge and hydrophobicity can be optimized to direct their localization in the sebaceous glands.^192^ The addition of ZnO, MgO, or CeO_2_ to such carriers can help stabilize particles, minimize aggregation, and co-load conventional actives (*e.g.*, retinoids, azelaic acid, niacinamide), which may reduce the amount of irritant molecules required.^193,194^ Nanofiber scaffolds fabricated by electrospinning or centrifugal spinning and loaded with ZnO or Ag/ZnO nanoparticles have also been proposed as acne dressings, exhibiting strong antibacterial activity, good breathability, and favorable mechanical properties.^195^

Combination therapies involving the co-delivery of MONPs with antibiotics or small molecule anti acne agents represent strategies that can be used to reduce the use of antibiotic and help mitigate antimicrobial resistance.^19^ ZnO and CuO nanoparticles have demonstrated synergistic effects with macrolides, tetracyclines, and organic acids, leading to reduced MIC values *in vitro*.^196^ In addition, microneedle and nanomotor platforms have been developed to co-deliver photosensitizers and zinc-based nanoarchitectures for combined chemo-photodynamic therapy.^197^ In the future, formulation development is likely to increasingly leverage computational tools and AI-driven optimization to balance particle size, drug loading, carrier composition, and release kinetics to maximize lesion targeting while minimizing disruption of the skin microbiome.^198^

## 6. Comprehensive toxicity, safety, and regulatory considerations

The safety of MONPs in acne therapy is primarily influenced by ROS-mediated cytotoxicity, release of metal ions, particle size, and surface coatings; therefore, they should be systematically evaluated using *in vitro*, *ex vivo* and *in vivo* models.^11^ The reactive surfaces of NPs can generate ROS that induce lipid peroxidation, DNA strand breaks, and mitochondrial dysfunction in keratinocytes and fibroblasts, particularly in the case of highly reactive oxides such as ZnO or CuO.^199^ A recognized mechanism contributing to toxicity is the “Trojan horse” effect, in which nanoparticles are internalized by cells, dissolve intracellularly, and generate high local concentrations of metal ions, especially Zn^2+^ and Cu^2+^.^200^ In contrast, CeO_2_ nanoparticles can function as antioxidants or controlled ROS generators, depending on their redox state and microenvironment, offering the possibility of modulating oxidative stress rather than simply amplifying it.^201^

Typically, keratinocytes (HaCaT), fibroblasts, and immune cell assays are used to quantify viability, oxidative stress markers, and cytokine release *in vitro*. Increasingly, three-dimensional reconstructed human epidermis and *ex vivo* human skin explant models are used, as these models more closely resemble barrier complexity and follicular microanatomy.^202^ For acne treatment, models that include inflamed or barrier-disrupted skin with intact pilosebaceous units are relevant, as local inflammation can increase ion permeation while also influencing the immune response to MONPs.^203^

The regulatory frameworks for nanomaterials in cosmetics and pharmaceuticals continue to evolve.^204^ Under the EU Cosmetics Regulation (EC No. 1223/2009, as amended), nanomaterials must be explicitly labelled and undergo thorough safety assessment, while cosmetic products containing nanomaterials must be notified through the Cosmetic Products Notification Portal (CPNP^205^). ZnO and TiO_2_ NPs have been extensively evaluated by the Scientific Committee on Consumer Safety (SCCS), and their use is subject to specifications regarding particle characteristics and concentration limits.^77^

In the United States, the U.S. Food and Drug Administration (FDA) expects drug products and over-the-counter (OTC) formulations containing nanomaterials to meet established standards for safety, efficacy, and quality, with an additional emphasis on physicochemical characterization, systemic exposure assessment, and long-term safety data.^206^ Approved iron oxide and gold-based nanomedicines provide precedents for the regulatory evaluation of inorganic nanomaterials.^207^

In the EU, only ZnO and TiO_2_ among the oxides discussed have been formally assessed for dermal use. The SCCS concluded that nano-ZnO and nano-TiO_2_ were considered safe (or of no safety concern) as UV filters at up to 25% concentration in sunscreens on intact or sunburnt skin.^208,209^ The SCCS safety conclusions did not extend to sprayable formulations because of concerns regarding inhalation exposure, and noted that nano-TiO_2_ carries a category-2 CMR classification by the inhalation route under EU CLP, although this classification was subsequently annulled by the Court of Justice of the European Union in August 2025.^210,211^

Two important considerations apply to acne products. These opinions cover the use of nanomaterials as UV-filter on largely intact skin. Daily application to inflamed, barrier-compromised acne-prone skin falls outside this scope. The same is true for the use of oxides as active antibacterials rather than UV filters. Both applications fall outside the scope of current assessments and would require their own safety dossier, with attention to ion release under low follicular pH. All EU nanomaterial cosmetics must also be notified through the CPNP and labelled. Safety must be assessed under Regulation (EC) No 1223/2009 as well.^210,211^

In the United States, the FDA has proposed that only ZnO and TiO_2_ be treated as generally recognized as safe and effective among sunscreen UV filters.^212^ Its nanotechnology guidance requires physicochemical characterization, systemic exposure assessment, and route-specific toxicology for nanoscale drugs and OTC products.^213^ Approved iron oxide nanomedicines provide a precedent for evaluating inorganic particles; however, no metal oxide is currently approved for acne treatment.^214^

Several other metal oxides, including CuO, Fe_3_O_4_, MgO, CeO_2_, MnO_2_, and Al_2_O_3_, fall outside the current regulatory assessments available for ZnO and TiO_2_. Consequently, these face a heavier regulatory burden for acne indications, and chronic metal-leaching data will be central to any regulatory submission.

Green synthesis can reduce residual toxic reagents, thereby minimizing exposure to potentially harmful contaminants.^215^ Biocompatible coatings such as polysaccharides, phospholipids, PEG, or protein coronas are used to mask the reactive surface and regulate ion release.^216^ To minimize deep penetration, delivery-system design strategies have been employed, including microneedles that can target follicular depots.^217^

Ultimately, the successful clinical translation of MONP-based acne therapies will require harmonized, multi-tiered toxicity testing that includes chronic exposure assessment, biodistribution studies, and evaluation of the effects on the skin microbiome, in alignment with emerging international regulatory science for nanomedicine.^21^ The comparative toxicity profiles and dermal safety considerations of the representative MONPs are summarized in Table 4.

**Table 4:** Comparative toxicity profiles and dermal safety considerations of representative metal oxide nanoparticles (MONPs)

Metal oxide nanoparticle	Key toxicities	*In vitro* safety	Safety record	References
Zinc oxide (ZnO NPs)	Dose and time-dependent keratinocyte cytotoxicity; TNF-α induction *via* an ROS- ERK-Egr-1 pathway; reversible collagen reduction at low dermal dose in rats	No irritation in a 3D reconstructed human epidermis model at 1 mg mL^−1^ after 24 h; cytotoxicity is dose- and time-dependent but comparable to non-nano ZnO	No penetration beyond the stratum corneum; a 90 days repeated-dose dermal study in rats found no observed adverse effect up to 1000 mg kg^−1^	218–221
Titanium dioxide (TiO_2_ NPs)	ROS-mediated oxidative stress in internal organs; sub-lethal developmental/physiological effects in aquatic organisms (zebrafish); inhalation, ingestion, and dermal exposure routes all documented, with inhalation the principal occupational concern	Stratum corneum functions as an effective barrier; sunscreen-formulated nano- and submicron TiO_2_ show minimal penetration into viable epidermis	UVB-damaged (sunburned) skin does not significantly enhance penetration *in vitro* or *in vivo*, supporting topical safety; environmental/aquatic toxicity remains a separate concern	222–225
Iron oxide, magnetite (Fe_3_O_4_ NPs)	Dose–dependent toxicity, variable with particle size, shape, and cell type; the IV contrast agent ferumoxytol carries an FDA boxed warning for fatal hypersensitivity reactions	No cytotoxicity in HaCaT keratinocytes at 5, 10, or 25 µg mL^−1^ after 24 h (Alamar Blue assay)	Acute dermal toxicity testing in hairless mice showed no significant impairment of skin barrier function; systemic hypersensitivity risk is specific to the IV route, not topical use	226
Copper oxide (CuO NPs)	Dose and time-dependent keratinocyte cytotoxicity; membrane damage; ROS generation; caspase-3-mediated apoptosis; genotoxicity increasing with dose, most pronounced at 20 µg mL^−1^	Marked cytotoxicity in HaCaT keratinocytes at concentrations as low as 20–30 µg mL^−1^, with concurrent oxidative stress and DNA damage	Highest relative toxicity among the oxides reviewed; particles 30–100 nm are internalized into the cytoplasm and nucleus, while particles >500 nm remain extracellular-underscoring the need for exposure control	227 and 228

Long-term dermal exposure is the most relevant scenario for acne because products are applied daily for months. The available human data are reassuring only for ZnO and TiO_2_, and only in the context of sunscreen. Intact particles remain in the stratum corneum and follicular infundibulum, and the zinc detected in the viable epidermis is present as dissolved ions rather than as particulate ZnO, without systemic accumulation at cosmetic use concentrations.^42^ Comparable chronic dermal datasets do not exist for CuO, MnO_2_, CeO_2_ and Al_2_O_3_. This evidence gap should be explicitly acknowledged rather than inferred from short-term assays.

The bioaccumulation potential differs significantly among oxides and is largely determined by their solubility. Sparingly soluble oxides such as TiO_2_, Al_2_O_3_, and, to a lesser extent, CeO_2_ and Fe_3_O_4_ raise the question of particle persistence. This is why the chronic concern for aluminium is systemic accumulation rather than acute toxicity and why iron oxide is evaluated against its established biodistribution as a contrast agent.^139,165^ Soluble oxides such as ZnO, CuO, and MgO instead raise an ion-burden question. The relevant quantity in this case is the local and systemic load of Zn^2+^, Cu^2+^ or Mg^2+^ relative to normal tissue levels.

Effects on the skin microbiome represent a distinct risk that has largely not been evaluated. The same broad antibacterial action that suppresses *Cutibacterium acnes* can perturb commensals, and we note the lack of before and after microbiome data as a specific gap.

The presence of TiO_2_ and ZnO NPs in aquatic ecosystems is known to cause subacute physiological alterations in aquatic organisms. These findings highlight the need to adopt green synthesis alongside material recovery protocols and comprehensive life cycle assessments directly at the product design stage. Because these environmental and processing considerations vary significantly across different metal oxides, an oxide-specific comparison is detailed in Table 5.

**Table 5:** Oxide-specific chemical stability, dermal fate and safety

Oxide	Solubility/ion release	Skin penetration	Accumulation/systemic risk	Redox behaviour	Main dermatologic concern	Acne implication
ZnO^229,230^	pH-dependent dissolution releasing Zn^2+^, with dissolution accentuated on acidic or inflamed skin	Confined to the stratum corneum and follicular infundibulum; particles do not enter the viable epidermis, but ionic Zn^2+^ liberated from the particle surface does	No evidence of systemic Zn accumulation at cosmetic use levels; SCCS has cleared nano-ZnO for dermal use up to 25% in sunscreen formulations	Mild ROS generation through Zn^2+^-mediated enzyme inhibition; photoactive under UV exposure	Keratinocyte cytotoxicity at high local dose; delayed wound closure reported *in vitro*	Strongest human evidence base among the oxides reviewed; a microbiome-sparing profile is achievable with dose and coating optimisation
TiO_2_ (ref. 77 and 223)	Essentially insoluble, with negligible ion release	Remains on the stratum corneum; penetration to viable skin is negligible even on UV-damaged skin	Low dermal accumulation; inhalation is the principal hazard, with TiO_2_ classified IARC group 2B and carrying an EU CMR category-2 (inhalation) hazard designation	UV-triggered photocatalytic ROS generation, markedly greater for the anatase than the rutile crystal phase	Phototoxic ROS generation in viable layers when uncoated; unsuitable for aerosolised/spray formats	Requires surface coating or a photo stabiliser to control photocatalytic activity as a UV filter
CuO^227,228^	Rapid, pH- and surface-area-dependent Cu^2+^ release, substantially exceeding that of micron-scale CuO	Particles in the 30–100 nm range are internalised by keratinocytes *via* endocytosis, whereas particles above roughly 500 nm are largely excluded	Local cyto- and genotoxicity at low µg mL^−1^ concentrations, with a narrow margin between antibacterial and cytotoxic doses	Cu^2+^/Cu^+^ redox cycling supporting Fenton-like hydroxyl radical generation	Keratinocyte cytotoxicity and genotoxicity, including nuclear copper accumulation and DNA strand breaks	Potent activity against *Cutibacterium acnes* but justifiable only with capped or release-controlled formulations
Fe_3_O_4_/γ-Fe_2_O_3_ (ref. 214)	Low solubility with slow, pH-dependent Fe release	Limited penetration when surface-modified with a hydrophilic coating	Clinically precedented biodistribution *via* ferumoxytol (an FDA-approved iron oxide nanoparticle formulation); rare hypersensitivity reactions are a recognised systemic caution	Peroxidase and catalase-mimetic nanozyme activity; Fenton-type chemistry favoured under acidic or NIR-activated conditions	Oxidative stress at high local dose	NIR/Fenton-activatable depot strategy of interest for recalcitrant nodular lesions
MgO^231^	Dissolves to Mg^2+^ and surface hydroxide, producing a locally alkaline environment	Low	Well tolerated, with a wide margin of safety reported in biomedical use	Surface basicity contributes to ROS-mediated action	Irritation if surface basicity or dose is not controlled	pH-buffering, sebum-adsorbing co-active rather than a primary antibacterial agent
CeO_2_ (ref. 183)	Low solubility with a redox-state-dependent surface	Low when adequately coated	Ultrasmall, surface-engineered particles show more efficient systemic clearance, addressing a key translational barrier for this oxide	Reversible Ce^3+^/Ce^4+^ cycling confers SOD- and catalase-mimetic, self-regenerating antioxidant activity that can switch to a pro-oxidant mode at higher local concentrations	Dose–dependent pro-oxidant switch	Redox-modulating, anti-inflammatory adjuvant rather than a direct antibacterial agent
MnO_2_ (ref. 10)	Reactive; catalytically decomposes H_2_O_2_ to O_2_ within the acidic biofilm niche, releasing Mn in the process	Delivered *via* a microneedle platform that bypasses the stratum corneum for direct follicular/dermal targeting	Limited standalone dermal accumulation data; evidence to date derives chiefly from composite nanomotor/microneedle systems	Catalase-mimetic O_2_-generating activity, acting as a Fenton-chemistry partner within composite platforms	Oxidative stress if catalytic activity is over-driven; sparse standalone safety data	Catalytic O_2_-generating component that relieves biofilm hypoxia around *Cutibacterium acnes* within composite microneedle platforms
Al_2_O_3_ (ref. 232 and 233)	Very low solubility, particularly for the α-phase, with minimal Al^3+^ release	Low	Chronic systemic aluminium accumulation, rather than acute toxicity, is the key concern flagged across pharmaceutical, occupational and consumer exposure routes; oral *in vivo* data show a modest, tissue-restricted genotoxic signal	Low ROS generation, especially for the α-phase	Genotoxic potential at high dose	Adjuvant/rheology modifier only; no direct evidence supports activity against *Cutibacterium acnes*

## 7. Clinical translation, challenges, and commercialisation

The translation of MONP-based acne therapies from the laboratory to the clinic is impeded by several scientific, regulatory, and commercial challenges.^21^ Although preclinical studies (mostly performed *in vitro* and using animal models) have shown the potential of ZnO, CuO, Fe_3_O_4_, MgO, and CeO_2_ systems in terms of antibacterial, antibiofilm, anti-inflammatory, and photothermal activity, comprehensive clinical data in humans are limited and mostly focus on systems containing ZnO, such as nanogels and sunscreen formulations.^234^ A recent systematic review of topical ZnO NP formulations concluded that nanoscale reformulation has a significant positive effect on the anti-inflammatory activities of zinc but also highlighted the lack of properly powered, randomized acne studies.^11^

No completed and adequately sized randomized controlled trials have investigated MONPs for acne from a clinical perspective. The only human-level evidence is for ZnO nanogels in small, early phase, and exploratory studies, and the systematic review by Crainic *et al.* reached the same conclusion while emphasizing the absence of properly powered acne trials.^11^ Evidence for CuO, Fe_3_O_4_, and MgO is currently limited to preclinical studies and should not be interpreted as evidence of clinical efficacy. The same holds true for CeO_2_, MnO_2_, and Al_2_O_3_. However, their use is explicitly limited to *in vitro* and animal models.

In addition to therapeutic efficacy, three specific translational challenges must be addressed in dermatological nanomedicines. Manufacturing and scale-up require reproducible control of the size distribution, morphology, aggregation state, and surface coating to pharmaceutical-grade tolerances. Scalable manufacturing is feasible using sol–gel, hydrothermal, or co-precipitation methods. However, these methods require careful control of process-related impurities, removal of residual solvents, and robust in-process quality controls.^235,236^

Green routes improve sustainability but trade this benefit for batch-to-batch variability, which is difficult to reconcile with GMP. Standard assays cannot resolve key therapeutic parameters, such as the ion-release rate, defect density, and photocatalytic flux, which require specialized nano-specific characterization methodologies for quality control. Cost scales with complexity: simple oxide dispersions can be economical, but multicomponent constructs such as Janus-nanomotor microneedle patches carry a manufacturing cost that will confine them initially to prescription or premium segments.^237^ Realistic near-term translation therefore favors simpler ZnO or CeO_2_ based leave-on and spot formulations, with the more elaborate activated platforms reserved for recalcitrant diseases, where their added cost is justified.

Moreover, the differences between tightly controlled experimental models (*e.g.*, biofilm monolayers and *ex vivo* skin) and the marked heterogeneity of acne lesions across the face, chest, and back complicate clinical translation.^21^ Data on dose selection, penetration depth, and long-term safety are difficult to extrapolate from murine models, which only partially recapitulate human follicular architecture and the complexity of the skin microbiome.^238^

Many studies refer to microbiome-centric solutions, including probiotics, bacteriophages, and peptide-decorated NPs with organic or polymeric cores,^238–240^ which provide blueprints for designing solutions based on MONPs (*e.g.*, peptide-targeted ZnO NPs that bind specifically to *Cutibacterium acnes* and kill them by ROS production and UV protection).^108^

Beyond manufacturing and cost, the current market trends favor non-antibiotic acne treatments that focus on the microbiome, with minimal irritation and no risk of resistance, providing a strong commercial rationale for MONP technologies that provide anti-inflammatory, sebum-modulating, and photoprotective properties, while also offering favorable cosmetic benefits such as a matte finish and minimal whitening.^241^ To achieve these goals, next-generation MONP platforms are expected to feature several high-end design features. This includes oxide cores with carefully controlled redox properties, such as zinc oxide (ZnO), iron oxide (Fe_3_O_4_), and cerium oxide (CeO_2_), which enable controlled ROS generation and ion release to enhance therapeutic effects.^242^ Additionally, the use of surface capping layers derived from biopolymers or generated through environmentally benign synthesis methods may further improve the biocompatibility, safety, and environmental friendliness of these nanoplatforms.^243^ In addition, strategies based on peptide-coated nanoparticles and bacteriophages, which are designed to be selective for the target and not affect the normal microbiota of the skin, are likely to help develop safer and more effective acne treatments.^244–246^ Advances in computational modelling of NP-skin interactions and AI-driven formulation optimization may further accelerate candidate selection and de-risk clinical programs by predicting penetration, efficacy, and toxicity profiles prior to large-scale trials.^247^

In conclusion, MONPs are unlikely to replace systemic antibiotics in the near future; however, they hold considerable promise as non-antibiotic, microbiome-friendly adjuncts or alternatives for topical acne therapy, particularly in mild-to-moderate acne and maintenance treatments.^248^ The convergence of green synthetic chemistry, smart delivery systems, and rigorous clinical evaluation will be critical for translating the compelling preclinical evidence supporting MONP-based platforms into commercially successful acne treatments that achieve regulatory approval.^21^

## 8. Future prospects

The multidimensional antibacterial, anti-inflammatory, antioxidant, and antibiofilm properties of MONPs are expected to be significant in the future management of acne vulgaris. Of the various systems studied, NPs of ZnO, CuO, TiO_2_, Fe_3_O_4_, MgO, and CeO_2_ have shown great promise as non-antibiotic alternatives that can target a multitude of pathogenic mechanisms of acne. Future studies are anticipated to be directed towards optimizing the physicochemical characteristics of MONPs, such as particle size, surface charge, morphology, and controlled release of ions, to increase follicle targeting, effectiveness against *Cutibacterium acnes* and overall skin compatibility. In addition, the safety, stability, and therapeutic activity of MONPs in dermatology are likely to be improved with the help of green synthesis approaches and biocompatible surface modifications.

Moreover, advanced MONP-based delivery systems, such as nanogels, hydrogels, microneedles, electrospun nanofibers, and light-responsive nanoplatforms, could significantly improve targeted drug delivery and sustain therapeutic effects in acne lesions. The TiO_2_, Fe_3_O_4_, and MnO_2_ NPs in photodynamic and photothermal modes can also be used for targeted killing and disruption of bacterial biofilms. However, important challenges related to long-term safety, large-scale manufacturing, regulatory approval, and clinical validation must still be addressed before these technologies can be translated into routine clinical practice.

Looking ahead, four areas are worth focusing on while keeping expectations realistic. AI-assisted design: machine learning models trained on particle descriptors such as size, coating, zeta potential, band gap, ion-release rate, skin penetration, or antibacterial outcomes could prescreen candidates and reduce the experimental search space before scale-up, and early work has already applied computational optimization to nanoparticle skin interaction.^198,247^ Precision and personalized dermatology would layer patient-level information, such as sebum output, acne severity, and subtype, and eventually follicular microbiome composition onto formulation choice, for example, matching a redox-modulating CeO_2_ adjuvant to oxidatively driven inflammatory acne and a photo-activated system to biofilm-heavy lesions. Microbiome-directed nanomedicine is the most distinctive opportunity and the least mature. Peptide or phage-decorated oxide cores intended to bias activity towards *Cutibacterium acnes*, evaluated by sequencing-based microbiome endpoints rather than colony counts, would move the field from ‘broadly antibacterial’ to ‘pathogen-directed’.^239,240,244,248^

Finally, rational combination with established acne actives (retinoids, azelaic acid, niacinamide, and salicylic acid) co-loaded in the same oxide-stabilized carrier may lower the dose of irritant molecules while retaining efficacy.^193^ None of these removes the need for the safety assessments and clinical trials outlined above, but together, they define a credible development path. Therefore, further studies should focus on detailed safety evaluations and carefully designed clinical trials to validate the efficacy and clinical safety of MONPs for treating acne vulgaris.

## 9. Conclusions

MONPs represent a promising non-antibiotic strategy for the management of acne vulgaris because of their ability to simultaneously target multiple pathogenic pathways, including *Cutibacterium acnes* colonization, follicular hyperkeratinization, excess sebum production, biofilm formation, and inflammation. Their multifunctional antibiofilm, antioxidant, anti-inflammatory, and photoresponsive properties, combined with advances in formulation approaches such as hydrogels, lipid carriers, nanofibers, microneedles, and nanomotor-based systems, have expanded their therapeutic potential beyond that of conventional acne treatment. However, successful clinical translation requires a comprehensive understanding of dermal fate, long-term safety, ion release kinetics, and nanoparticle–host interactions. In addition, standardized manufacturing processes and regulatory harmonization are important aspects. Future research should focus on microbiome-conscious design, scalable and reproducible synthesis, and robust clinical evaluation to establish the safety, efficacy, and translational feasibility of MONP-based therapies. Overall, MONPs offer considerable promise as next-generation therapeutic platforms for acne vulgaris and warrant further investigation towards clinical applications.

## Author contributions

Akhil Nair: conceptualization, methodology, investigation, data curation, formal analysis, validation, visualization, writing – original draft, writing – review and editing. Sindhoor S M: conceptualization, methodology, investigation, data curation, formal analysis, validation, resources, visualization, writing – original draft, writing – review and editing. Shravya D Rai: conceptualization, methodology, investigation, data curation, formal analysis, validation, resources, visualization, writing – original draft, Writing – review and editing. Gladyston Netto: conceptualization, methodology, investigation, data curation, formal analysis, project administration, supervision, funding acquisition, resources, writing – review and editing. Srinivas Mutalik: conceptualization, methodology, investigation, data curation, formal analysis, project administration, supervision, resources, writing – review and editing. Pavithra Pradeep Prabhu: conceptualization, methodology, investigation, data curation, formal analysis, project administration, supervision, resources, writing – review and editing.

## Conflicts of interest

There are no conflicts to declare.

## Data Availability

No primary research results have been included, and no new data were generated or analysed as part of this review.
